# Assessment of the Carrying Capacity and Suitability of Spatial Resources and the Environment and Diagnosis of Obstacle Factors in the Yellow River Basin

**DOI:** 10.3390/ijerph20043496

**Published:** 2023-02-16

**Authors:** Yu Chen, Shuangshuang Liu, Wenbo Ma, Qian Zhou

**Affiliations:** 1 School of Economics and Management, Zhengzhou University of Light Industry, Science Avenue 136, Zhengzhou 450000, China; 2 School of Management, Henan University of Technology, Lianhua Street 100, High-Tech Zone, Zhengzhou 450000, China; 3 Economics School, Zhongnan University of Economics and Law, Nanhu Avenue 182, Wuhan 430073, China

**Keywords:** production–living–ecological space, national land space, carrying-capacity evaluation of resources and environment, suitability evaluation, barrier degree

## Abstract

The assessment of the carrying capacity and suitability of spatial resources and the environment can provide effective guidance for regional planning and make vital contributions to the high-quality advancement of society and the economy. Additionally, this scientific evaluation of the spatial carrying capacity and suitability of urban production–living–ecological space (PLES) has important scientific value as well as practical significance for territorial spatial planning. This paper takes the cities along the Yellow River Basin (YRB) as the research object; establishes the PLES resource and environment carrying capacity evaluation index system; uses the multi-indicator superposition method and entropy weight method to evaluate the ecological importance, production and life carrying capacity of 78 cities in the YRB from 2010 to 2020; obtains the final ecological importance, production and life suitability levels based on the carrying capacity combined with the location conditions; and uses exploratory spatial data analysis (ESDA), the barrier degree model and other methods to determine the cities’ spatial and temporal patterns and influencing factors. The findings indicate that: (1) Ecological importance is characterized by “high upstream and low downstream”; the suitability for production is higher mainly in the eastern coastal area; the suitability of life as a whole is increasing, and the higher grade is in some provincial capitals and surrounding cities. (2) The local Moran’s I all passed the test, and the relationship between PLES showed a significant positive spatial correlation. The clustering characteristics of ecological importance and production suitability are strong, while the clustering characteristics of suitability for living functions are relatively weak. (3) Biodiversity, significance of water conservation and significance of wind and grit control functions are the main barrier factors affecting the ecological importance of the YRB; industrial value-added output per unit of industrial land, number of industrial enterprises above the scale and GDP per capita are the dominant factors affecting the production suitability of the study area; total water-resource utilization per capita, total sewage treatment per capita and residential land area per capita are the main barrier factors of living suitability.

## 1. Introduction

The national land space is a valuable natural resource of the state and the nation, and is the spatial carrier for the nation to carry out production, living and ecological civilization construction. However, rural populations have generally declined in countries around the world, and the hollowing out of people first emerged with the urbanization and industrialization process of recent years. Over the past forty years or so, China’s rate of urbanization has increased from 17.92% in 1978 to 64.72% in 2021, and the rapid development of urbanization and rapid industrialization has caused a strong squeeze on ecological space and agricultural production space [1], creating a number of issues, including the loss of water and soil resources, the ruination of the ecological environment and the deterioration of human quality of life, which has intensified the competition between ecological areas, production areas and living areas. The competition between ecological space, production space and living space has intensified, leading to land use conflicts between PLES [2]; this conflict, further, causes contradictions in the use of land and space to intensify. The construction of a scientific and reasonable spatial development pattern of the territory of China and the optimization of the land use layout of PLES are important references for addressing the issue of international hollowing out, and are relevant and significance in the long term for the comprehensive construction of a modern socialist country in the new era and new development stage. For this reason, the 18th National Congress of the Communist Party of China, for the first time, specified the development requirement of “promoting intensive and efficient production space (PS), livable and moderate living space (LS), and beautiful ecological space (ES)”. The Third Plenary Session of the 18th CPC Central Committee proposed to “establish a spatial planning system, delineate the boundaries of production, living and ecological space development control, and implement use control”. In 2019, “Several Opinions on Establishing Territorial Spatial Planning System and Supervising Its Implementation” made it clear that the “scientific layout of PS, LS, ES is a key initiative to build a beautiful China, is to take a people-centered approach, the construction of a beautiful home important measures”. Therefore, there are many studies on the evaluation of the main function of national space and its adjustment and optimization. Additionally, the development of an evaluation system that can reflect the philosophy of high-quality advancement and the search for obstacle factors according to the evaluation results have become the key to research. The document titled “Outline of the YRB’s Ecological Protection and High-quality Development Plan”, issued by The Communist Party of China Central Committee and the State Council in 2021, proposes comprehensively assessing the resources and environmental carrying capacity of the YRB, coordinating the needs of the PLES in the territory of China, and evaluating the suitability of territory development to form a pattern that uses the territory of China efficiently and rationally develops the territory of China. Based on this, this paper takes cities along the YRB as an example and constructs an evaluation index system of ES importance, suitability of LS and production suitability from two dimensions of water and soil resources carrying capacity and development suitability, taking into account the natural geography, society and economy of the YRB. This study assessed the status and suitability level of soil and water-resource use in the YRB and identified the geographic distribution pattern of PLES and the obstacle factors with the help of an obstacle degree model. On this basis, the functional zoning of land space utilization is optimized to offer scientific reasons for the ecological management of the YRB and the rational planning of territory.

## 2. Literature Review

### 2.1. Spatial Identification and Conflict Diagnosis of PLES

PLES was developed from agriculture’s versatility, which was initially proposed in the Uruguay Round Agreement on Agriculture (URAA) [3], and academics have further extended multifunctionality into land use functions as well as landscape functions [2,4]. In the SENSOR (Sustainability Impact Assessment: Tools for Environmental Social and Economic Effects of Multifunctional Land Use in European Regions) program, land functions were classified into the social, economic and ecological [5]. According to this, Chinese researchers combined Chinese national conditions and the overall plan for land utilization and then divided the territory into PS, LS and ES according to its dominant functions [2].

PLES focuses on identification and classification, spatial conflict, and simulation and prediction. In terms of identification methods, the identification methods of PLES are mainly divided into the top-down indicator measurement method and the bottom-up land-use-type method, with the former evaluating the land use function by constructing an indicator system using the coupled coordination degree model [6], the entropy-value method [7], and the GIS spatial analysis method [8,9] and dividing the PLES by the evaluation results; the latter merges and divides the spaces with equally dominant functions based on land use types [10,11]. The conflict of the PLES is essentially the result of the need to make trade-offs between the quantity of land and its usage because of the limited amount of territory and the constraints of the climatic environment [12], which, in turn, leads to conflicts, manifested in the process of spatial and temporal competition and gaming between various land use subjects in the same location for spatial resources with land as the core [13,14]. Research on land use conflicts is divided into conflict identification [15], spatial evolution [11], and drivers [16,17]; thus far, as far as scenario simulation is concerned, theories and methods for simulating land use changes based on various scenarios have gradually matured with the advancement of land use research [1]. Different research methods have different applicability and characteristics, and simulation models are broadly divided into quantitative forecasting models and spatial pattern forecasting models. Among the quantitative forecasting models, the main ones used are SD models [18], Markov models [19,20], and GM models [14,21]; regarding spatial-pattern forecasting models, the main ones are ANN-CA [22], CLUE-S models [23], and FLUS [2,24,25].

### 2.2. Assessment of the Carrying Capacity and Suitability of Spatial Resources and the Environment

The evaluation of PLES is a crucial foundation for improving the spatial development of national land [26], and it is mainly divided into the suitability evaluation [27] and carrying-capacity evaluation of resources and environment [28,29,30]. The research on PLES evaluation focuses on evaluation methods and indicator systems, and the evaluation methods that researchers use more often in the research process are multifactor overlay comprehensive evaluation and spatial interaction. Comprehensive evaluation of multifactor overlay is based on spatial overlay, combining mathematical and statistical methods with GIS technology to develop different index-fitting algorithms [31,32]; since land use development is not only related to local resource endowment but also the natural environment and human actions can have a profound impact on spatial development [33], researchers began to evaluate it from the perspective of spatial interactions by constructing cumulative resistance models with the help of landscape pattern variables [34].

### 2.3. Conclusion

In summary, many researchers have researched the PLES from different perspectives and using different methods, which shows the diversification of research themes, rationalization of index construction and maturation of research methods, but there are still areas that need in-depth research. First, most of the studies on the evaluation of the PLES focus solely on the carrying-capacity evaluation of resources and the environment or the suitability of spatial resources and the environment; another key fact to remember is that there are few studies that combine the two. Second, most researchers adopt static evaluation in the process of evaluation, but resource and environment carrying capacity and suitability level are not constant, but experience dynamic change, and dynamic research is the inevitable way to scientifically recognize the evolution of a regional development pattern. Hence, this study takes the YRB as the study area, establishes a multilevel evaluation system based on the idea of PLES based on the traditional evaluation levels of ecology, agriculture and towns in the “double evaluation” system, and implements the relatively conceptualized concept of PLES into the “double evaluation” process through the screening of evaluation indices. From the perspective of PLES, examining the current situation of the spatial development of national land, analyzing its carrying function and the suitability of development, the concept of PLES is no longer a slogan but is actually reflected in the basic analysis of planning.

## 3. Methods and Data

### 3.1. Study Area

**Figure 1:**
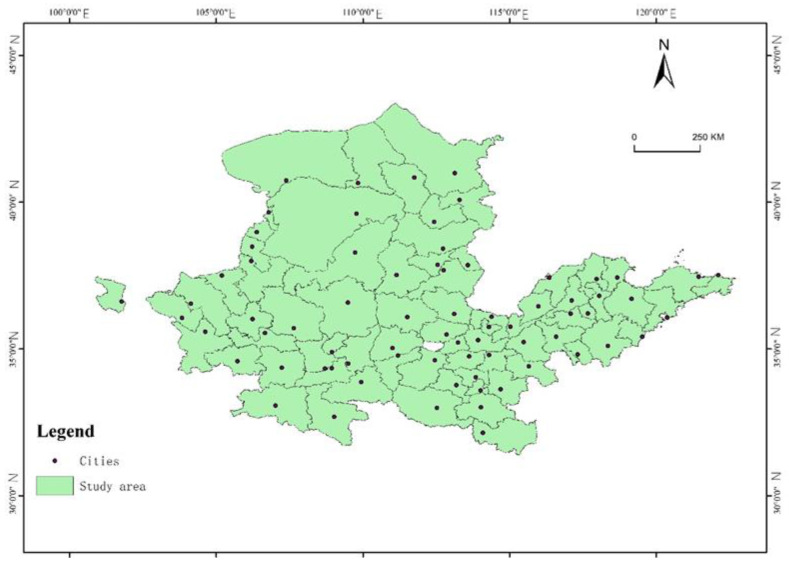
Sample area: the Yellow River Basin.

The Yellow River is located at latitude 31°31′ N~43°31′ N, longitude 89°19′ E~119°39′ E. The YRB originates in China’s Bayan Har Mountains of Qinghai Province, from west to east, flowing through Qinghai, Sichuan, Gansu, Ningxia, Inner Mongolia, Shaanxi, Shanxi, Henan, and Shandong, 9 provinces, and the Yellow River runs through the eastern, western and eastern regions of China. According to natural background conditions, the YRB is relatively rich in land resources, containing 15% of the country’s arable land, but the largest contradiction in the YRB is water shortage, meaning that water resources cannot meet the demand. Although the Yellow River is the fifth longest river in the world, the amount of river runoff is only 2.2% of the national amount of river runoff. The population in the YRB and its downstream irrigation areas mean that 12% of the country’s population use water from the Yellow River. Additionally, 67% of water resources have been developed and used in the YRB. This land for production and living is constrained by topography, water resources and other natural conditions. In terms of the social economy, the YRB’s entire advancements lag behind those of other regions, and there are some problems in regional development that are not balanced; conflicts between environmental protection and economic development are more severe. Generally, the YRB is limited by poor resources and the environment and unreasonable ways of utilizing territorial space, resulting in many problems, such as a fragile ecological environment, a lack of water resources in local areas, and the urgent need to enhance the status of land development. The study area is shown in Figure 1.

### 3.2. Research Methods

#### 3.2.1. Assessment of the Carrying Capacity and Suitability of Spatial Resources and the Environment

With reference to national regulations and existing academic research results, combined with the principles of regional specificity, data accessibility and index representativeness, this text used the model of single factor-integrated evaluation to evaluate the carrying capacity of resources and environment in ES, PS and LS around the elements of resources of the land as well as water, climate, natural disasters and socioeconomic factors. The current utilization level based on the shortcomings of the elements is constructed in this text, and the carrying capacity of the PLES is evaluated by measuring the carrying capacity for territory resources, water resources and nature. If development or construction activities in excess of the carrying capacity exceed the bottom line of resources and the environment, then the area is inevitably an unsuitable zone for production and life. The bottom-line restraint of resources and environment carrying capacity as suitability reflects “bottom line thinking” and the goal of forcing the transformation to high-quality development [35]. Therefore, this kind of evaluation is a comprehensive evaluation of the suitability of the PLES for the development of specific regional functions by combining socioeconomic development potential, location conditions, strategic orientation and other factors. Based on the carrying-capacity evaluation of resources and the environment, the regional spatial relationship is added, and the development suitability is used to further modify and supplement the carrying-capacity level so that the evaluation is closer to the objective reality.

**Figure 2:**
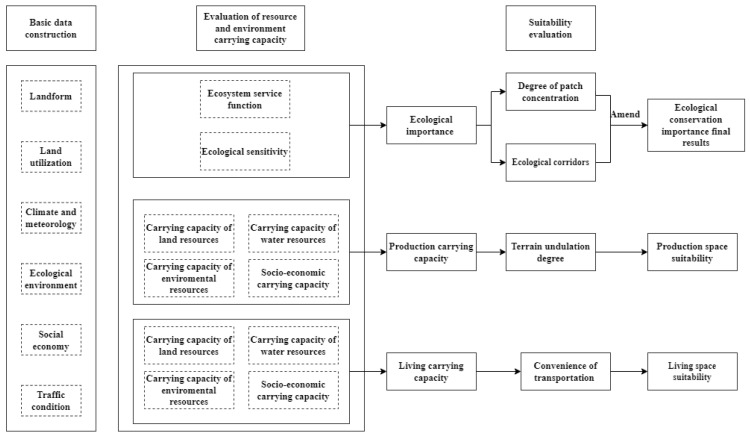
Technical process of resource and environment carrying-capacity assessment and suitability assessment.

This study takes the YRB as an example, and selects evaluation indexes from soil and water, climate, environment, socio-economic and location conditions from production, life and ecological perspectives to investigate the ecological importance and the maximum scale of production and life, and to analyze and evaluate the importance of ecological protection, the suitability of agricultural production and the suitability of urban construction, respectively. The technical roadmap is as follows (Figure 2).

#### 3.2.2. Spatial Pattern Analysis

ESDA is a more ideal data-driven analysis method. This method takes spatial correlation as the central concept and uses descriptions and illustrations of the spatial phenomena of objects to discover spatial anomalies and spatial clustering research objects intuitively to explore and study the interaction mechanism of spatial elements. ESDA analysis involves measurement and testing of global spatial autocorrelation and identification of local spatial association.

(1) Global spatial autocorrelation

Global spatial autocorrelation reflects the overall trend of the spatial correlation of observed variables in the whole study area, and the commonly used measurement index is global Moran’s I, which is used to explain the whole spatial correlation of the suitability of the land use of PLES.

(1) $$I=\frac{\sum_{i=1}^{n}\sum_{j=1}^{n}W_{ij}\left(X_{i}-\bar{X}\right)\left(X_{i}-\bar{X}\right)}{S^{2}\sum_{i=1}^{n}\sum_{j=1}^{n}W_{ij}}$$

In Equation (1), n is the number of study objects; $X_{i}$ and $X_{j}$ denote the observed values in regions, respectively; $W_{ij}$ is the spatial weight matrix ($W_{ij}$ was set according to whether region i and region j are adjacent: when region i and region j are adjacent, $W_{ij}=1$; when regions i and j are not adjacent, $W_{ij}=0$); $S^{2}$ is the variance of the observed attributes; and X is the mean of the observed attributes.

(2) Cold- and hot-spot analysis, $Gi^{*}$ index

$Getis-Ord\;G^{*}$ is used to identify the locations where significant high-value areas and low-value areas converge in space; therefore, it is used to analyse the local spatial autocorrelation characteristics of the spatial suitability of land use of PLES.

(2) $$G^{*}=\frac{\sum_{j=1}^{n}W_{ij}X_{i}}{\sum_{j=1}^{n}X_{j}}$$

In Equation (2), $W_{ij}$ is the spatial weight matrix; when region i and region j are adjacent, $W_{ij}=1$; when regions i and j are not adjacent, $W_{ij}=0$. If $Gi^{*}$ is positive and significant, the level of suitability around i is relatively high and it belongs to the hot-spot area; in contrast, if the level of suitability around i is relatively low, it belongs to the cold-spot area.

#### 3.2.3. Obstacle Degree Model

In this paper, the obstacle degree model is used to analyse the factors that hinder the suitability of PLES and is calculated from the factor contribution ($F_{j}$) and the indicator deviation $I_{ij}$, calculated as in Equations (3) and (4)

(3) $$I_{ij}=1-Y_{j}$$

(4) $$O_{j}=\frac{F_{j}I_{ij}}{\sum_{i=1}^{n}F_{j}I_{ij}}$$

where $I_{ij}$ is the index deviation degree; $Y_{j}$ is the standardized value of the indicator; $F_{j}$ is the factor contribution expressed by the weight of a single indicator ($W_{j}$); and $O_{j}$ is the obstacle degree.

### 3.3. Selection of Indicators

The evaluation was guided by Xi Jinping’s thought on ecological civilization and strictly followed the ecological priority principles and protection of arable land. Reference was also made to the “Guidelines for Evaluation of the Carrying Capacity of Resources and Environment and Suitability of Land Development (for Trial Implementation)” issued by the Ministry of Natural Resources of China in June 2019 and January 2020 and related research [36,37,38]. First, the importance of ecological protection was evaluated for the study area. Ecosystem service function refers to the benefits that human beings can obtain from the ecosystem for the purpose of survival, and these benefits are reflected in the conditions and processes of the ecosystem, including the intangible service function and tangible material supply in the ecosystem. Based on this, the importance of ecosystem services was measured by overlaying the functions of water conservation, conservation of water and soil, sand fixing and wind mitigation, and biodiversity maintenance. Ecological sensitivity refers to the potential intensity of the activity of an ecological process in an ecosystem under natural conditions and is used to indicate how sensitive it is to human activity; the higher the ecological sensitivity is, the higher the probability of causing ecological risk and the need to strengthen protection efforts. The evaluation factors of water- and soil-erosion sensitivity, sensitivity to land desertification and sensitivity to rocky desertification were selected, and the ecological sensitivity levels were classified through a GIS spatial weighting overlay. In this paper, the following factors were selected for inclusion in the evaluation system (Table 1), based on the aforementioned, combined with the characteristics of the geographical environment of the YRB (Table 1).

### 3.4. Data Sources

The spatial classification of the YRB is based on land use types, with vector data on administrative regions, DEM, NPP (net primary productivity), NDVI (normalized difference vegetation index), population density, spatial distribution of terrestrial ecosystem types and land use data. The data were collected from the Data Centre for Resource and Environmental Sciences of the Chinese Academy of Sciences. Meteorological data were collected from the National Center for Environmental Information (NCEI) of the National Oceanic and Atmospheric Administration (NOAA) and soil texture-type data from the Harmonized World Soil Database (HWSD). Economic and social statistics are collected from the China Urban Statistical Yearbook 2010–2020 and the National Economic and Social Development Bulletin of each municipality, of which water resources data are taken from the water resources bulletin of each province and municipality.

## 4. Results

### 4.1. Assessment of the Spatial Carrying Capacity and Suitability of the YRB of PLES

#### 4.1.1. Ecological Importance Assessment

**Figure 3:**
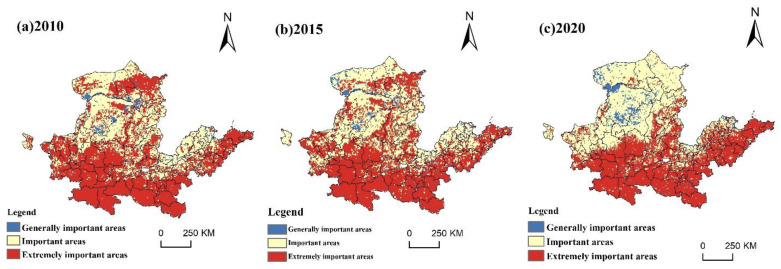
Spatial and temporal patterns of conservation levels of ecological importance from 2010 to 2020.

Data were processed based on ArcGIS 10.2 software, and the higher level of the significance of ecosystem service function and sensitivity of ecology was taken as the initial result of the ecological protection importance ranking. According to the criteria of the “Technical Guide for Carrying Capacity Evaluation of Resources and Environment and for Suitability of Territorial Area Development”, the importance of ecological protection will be divided into three parts: the importance of the general level, the importance of the higher level, and the importance of the highest level, and the spatial and temporal patterns of ecological-protection importance levels will be mapped for 2010, 2015 and 2020 (Figure 3).

**Figure 4:**
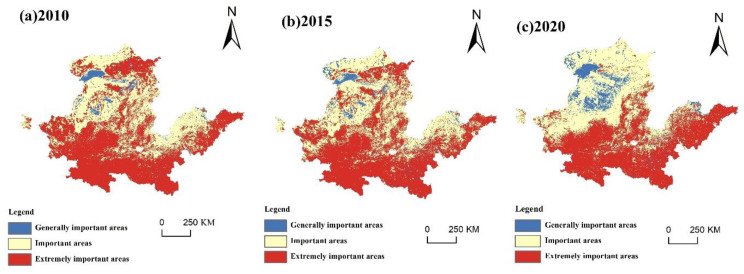
Spatial and temporal patterns of the revised protection levels of ecological importance from 2010 to 2020.

First, ecological patch concentration can portray the scale and spatial differentiation of ecological service functions and ecologically sensitive areas [39], so the ecological patch concentration is used as a correction factor. Ecological corridors are types of corridors with ecological service functions such as biodiversity protection, pollutant filtration, preventing loss of water and soil erosion, wind prevention and sand fixing, regulating floods and so on [40]. As an important ecological corridor in China, the Yellow River has a variety of ecological functions. Therefore, with reference to the “Guidelines for Evaluation of the Carrying Capacity of Resources and Environment and Suitability of Land Development (for Trial Implementation)” issued by the Ministry of Natural Resources of China in June 2019 and January 2020, the ecological patch concentration and the ecological corridor were used to correct the assessment level, and the results were obtained according to the discriminant matrix (Table 2), as in Figure 4.

In 2010, the level of ecological importance protection was very high, accounting for 57.75% of the entire region of the study area. The most ecologically crucial places were located in Tianshui, Baoji, Hanzhong, Ankang, Shangluo, Nanyang, Zhumadian, Xinyang, Zhoukou, Shangqiu, Qingdao, Weihai, Yantai and Ulanqab. The areas of high ecological-protection importance were mainly distributed in Bayannur, Ordos, Wuzhong, Yulin, Xinzhou, Anyang, Puyang, Dezhou, Jinan, Binzhou and Dongying. General important areas were mainly distributed in Bayannur, Ordos and Hohhot, but there were generally important areas scattered in a large number of cities. The ecological importance level was 3.28% of the entire region of the study area.

In 2015, highly ranked locations were scattered throughout the eastern and western halves of the research area, accounting for 55.74% of the entire region. The cities in the middle reaches of the YRB and cities such as Baoyannur, Baotou, and Ulanqab had an obvious decreasing trend. The generally important areas were scattered primarily in the north and northeast, the proportion reached 40.71%, and the overall trend gradually increased from northwest to southeast. The generally graded areas were mainly scattered in the north. Compared with 2010, there are fewer areas of this class scattered across cities. Many cities have reached the level of high ecological importance and above in all parts of the city, and the area of this level accounts for 3.55% of the entire study area.

In 2020, the areas of the ecological importance protection of the highest level were significantly reduced, and they were intensively concentrated in the southeast of the study area, accounting for 47.75% of the total area; important protected areas were mainly concentrated in the Inner Mongolia Plateau and scattered in Jining, Dezhou, Binzhou and other cities, accounting for 46.94% of the study area; areas with an importance of higher levels were mainly concentrated in Ordos, Yulin, Bayannaoer and other cities, accounting for 5.31% of the area. Overall, it shows a trend of increasing grade from the northwest to southeast of the YRB, and the extremely important protected areas appeared to agglomerate.

#### 4.1.2. Evaluation of Production Carrying Capacity and Suitability

(1) Evaluation of production capacity

**Figure 5:**
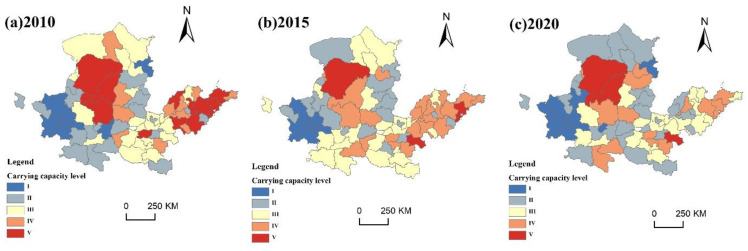
Spatial and temporal patterns of production capacity from 2010 to 2020.

According to the entropy method, the production-carrying-capacity index of each city was calculated. Using ArcGIS 10.2 software, Natural Breaks (Jenks, OK, USA) was used to divide it into Parts I, II, III, IV, and V, from low to high. The higher the level is, the higher the carrying capacity. For the higher level, the temporal and spatial pattern of production carrying capacity of prefecture-level cities in 2010, 2015, and 2020 was drawn (Figure 5).

The figure demonstrates that in 2010, the top-level and upper-middle-level urban production-carrying-capacity areas were mostly concentrated in the lower reaches of the YRB. The top-level production-carrying-capacity areas mainly included Ordos, Yulin, Yanan, Zhengzhou, Jining, Dezhou, Linyi, Weifang, Qingdao, and Yantai; the upper-middle-level areas mainly included Baotou, Luliang, Linfen, Pingdingshan, Zhoukou, Liaocheng, Jinan, Liaocheng, Tai’an, Dongying, and Zaozhuang; the intermediate-level production-carrying-capacity areas mainly included Baoyannur, Ulanqab, Hohhot, Taiyuan, Xi’an, Luoyang, Jiaozuo, Xinxiang, Kaifeng, Shangqiu, and Anyang; the lower production-carrying-capacity and lowest-level areas were primarily found in the northeast and southwest of China, where the research region is located; and the lower and lowest-level cities included Xining, Lanzhou, Baiyin, Guyuan, Xinzhou, and Datong. The overall level showed a gradual upwards trend from west to east.

In 2015, the upper middle level and top level areas were concentrated in the north-eastern and southern regions of the entire study region, accounting for 38.67% of the area. Top-level cities included Ordos, Shangqiu and Qingdao, the proportion of which was significantly reduced, the upper middle level areas showed expansion, and the urban carrying-capacity level of the lower YRB decreased significantly; the intermediate level was mainly distributed in the northeast, the Central Plains, and the northwest, and, compared with 2010, it showed an expansion trend; the places at the lower and lowest levels were mostly distributed in the upper YRB. The lower level urban areas gradually moved to the north, and the number of lower level urban areas was decreasing.

In 2020, the top-level and upper-middle-level areas accounted for 29.39%, and the area proportion decreased significantly. The top-level areas were mainly localized in Ordos, Yulin, and Shangqiu. The distribution of upper-middle-level areas was scattered and decreased; the south-eastern part of the study region was mostly where the intermediate level area was located. Around the middle reaches and downstream of the YRB showed a decreasing trend. The lower and lowest-level areas spread when compared to 2015, and the lower and lowest-level areas expanded. The carrying capacity of urban areas in the YRB’s lower reaches is significantly weakened.

(2) Production suitability evaluation

The suitability of production reflects the degree of the suitability of productive activities in territorial space. The evaluation results are divided into grades I, II, and III. The higher the grade is, the higher the suitability.

Alternative areas for different levels of suitability were determined according to the level of carrying capacity: an area suitable for the distribution of production space should first have comprehensive resources and environmental conditions for carrying production activities; the higher the carrying capacity of the environmental foundation and the lower the limitation of disaster risk, the higher the suitability of production. According to the production-carrying-capacity grade, the production suitable area and general suitable area of the alternative area were determined. Ensuring that the level of hazard is sufficient to meet basic production needs, areas suitable for the distribution of production space should have a certain level of flatness, and the greater the relief is, the greater the hazard; therefore, the relief was chosen as the correction factor, and the evaluation result is shown in Figure 5.

In 2010, the grade-III-production-suitable regions of the study area were concentrated in several cities in the eastern, central, and northern parts, mainly showing agglomeration distribution, accounting for 28.68% of the area. The second-level-production-suitable areas were mainly found in the central and northern regions, showing that the area around the cities of Ordos, Yulin and Yanan accounts for 62.3%. The majority of cities with Grade-I-acceptable areas are located in the western and northern regions, accounting for 11.02% of the area.

In 2015, the level-III-production-suitable area included Ordos in the central and northern parts and expanded to the southwest in the south, including Zhengzhou, Kaifeng, Heze, Jinan, Qingdao, Weihai and other cities. The distribution of the level-II-suitable-production areas expanded, and its area proportion was 68.04%. The area expansion was mainly due to the lower suitability grades of Yulin and Yanan and the higher suitability grades of Datong and Weinan. The level-I-suitable areas decreased, and their area accounted for 7.36%, including the cities of Dingxi, Baiyin, Guyuan, and Pingliang.

**Figure 6:**
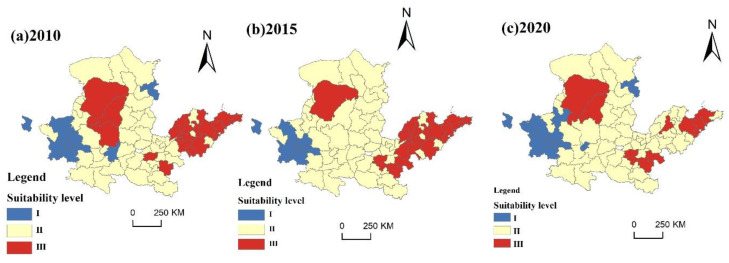
Spatial and temporal patterns of production suitability grades from 2010 to 2020.

In 2020, the areas suitable for level-III production decreased in the central and southern regions and expanded in the northern regions, accounting for 19.74%. The areas suitable for level-II production expanded to the southeast, while the level of suitability decreased in the south-eastern regions. The areas suitable for level-I production expanded, and the proportion was 11.78%, with some cities, including Datong and Tongchuan, downgrading their suitability for production (Figure 6).

#### 4.1.3. Living Capacity and Suitability Evaluation

(1) Evaluation of living carrying capacity

In 2010, the areas with higher living capacity levels and above were scattered, among which the level-V-carrying-capacity areas included Hanzhong, Ankang, Zhengzhou and Zhoukou, accounting for 5.80%. The level-IV-carrying-capacity cities included Baotou, Erdos, Wuhai, Shizuishan, Yinchuan, Xianyang, Shangluo, Jinan, Dongying, Qingdao, and Yantai, and the area accounted for 19.11%. The concentrated distribution area of level-III living capacity was mainly in the southeast and western Central Plains, including Ulanqab, Hohhot, Yulin, Xining, Xian, Baoji, Tongchuan, Nanyang, Luoyang, Jining, Weifang, Rizhao and other cities, accounting for 29.18%. The lower living capacity and lowest regions were mainly found in the west and around the Central Plains, showing a rise from west to east, including Lanzhou, Baiyin, Zhongwei, Linfen, Jincheng, Anyang, Puyang, Liaocheng, Dezhou, Binzhou and other cities.

In 2015, the level-V-capacity-region distribution was scattered, including Yinchuan, Xianyang, Zhengzhou, Zhoukou, and Ulanqab. The level-IV-carrying-capacity area expanded, including Ordos, Wuhai, Ankang, Yantai and other cities. Compared with 2010, the area of level-III living capacity was significantly reduced, accounting for 26.71%. The carrying-capacity zones of level I and level II were mainly found in the western and central plains regions, with the expansion of the lower level area and a reduction in the lowest level area.

**Figure 7:**
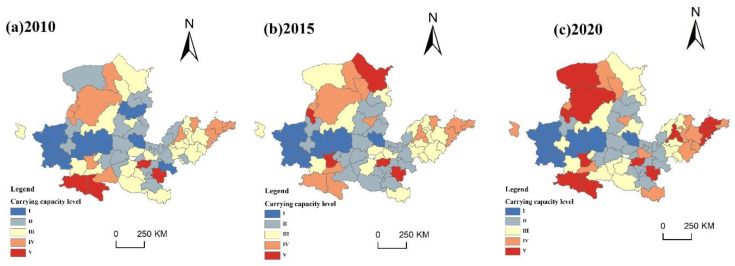
Spatial and temporal patterns of living carrying capacity from 2010 to 2020.

In 2020, most regions were found in the west and around the Central Plains, with level-V carrying capacity increasing significantly, accounting for 25.75%, and the southwest and east regions included Bayannur, Ordos, Yinchuan, Hanzhong, Ankang, Xianyang, Zhengzhou, Zhoukou, Jinan, Qingdao and Yantai. The level-IV carrying capacity decreased, and was mainly distributed in some cities in Shandong Province. The cities with level-III carrying capacity were further reduced, and the eastern and northern regions are where they are most prevalent. The carrying capacity of level I and level II is reduced. The carrying capacity of level I is not significantly different to that of 2015, while the carrying capacity of grade II is reduced in the southwest (Figure 7).

(2) Life suitability evaluation

**Figure 8:**
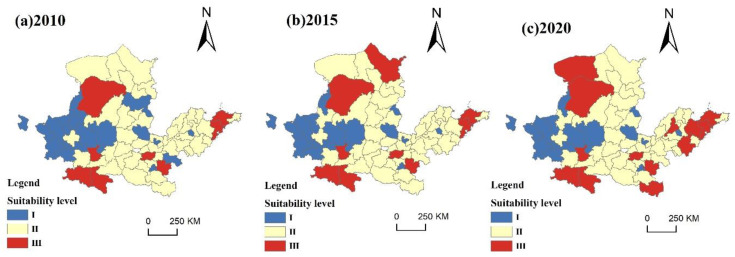
Spatial pattern of living suitability from 2010 to 2020.

The evaluation results of living suitability were classified into three main categories: suitable area, general suitable area and unsuitable area (Figure 8).

The degree of transportation convenience has a great influence on life suitability, which mainly represents the support and guarantee ability of road transportation facilities for life activities. Therefore, combined with data representativeness and availability, the highway mileage data of 2010, 2015 and 2020 were selected to represent the degree of transportation convenience. The larger the urban highway mileage data are, the higher the degree of transportation convenience.

In 2010, the distribution of level-III suitable areas was relatively scattered, including Erdos, Xianyang, Hanzhong, Ankang, Zhengzhou, Zhoukou, Qingdao and Yantai, occupying 23.08% of the research area. The middle and lower parts of the YRB, as well as the northern section, are where most of the level-II-appropriate regions are located, including Hohhot, Baotou, Taiyuan, Xian, Luliang, Linfen, Luoyang, Xinxiang, Heze, Jinan and other cities, accounting for 59.60% of the total area. The level-I-suitable areas are concentrated in the west and scattered in the central and eastern regions, including Xining, Lanzhou, Baiyin, Qingyang, Yanan, Xinzhou, Changzhi, Puyang, Shangqiu, Zibo and other cities, accounting for 23.08%.

In 2015, the spatial pattern did not change significantly. The level-III-suitability area increased, and the level of suitability in Ulanqab increased from level-II suitability in 2010 to level-III suitability. The suitable area for level II showed a decreasing trend, accounting for 58.80% of the area. The suitable area of level I shrank to the west as a whole, accounting for 18.24%.

In 2020, the level-III-suitability areas were further expanded, with a westward expansion in the Shandong Peninsula urban agglomeration, accounting for 29.16%. The suitable area for level II was further reduced, accounting for 52.60%. The spatial pattern of the level-I-appropriate area remained unchanged.

### 4.2. Spatial Agglomeration Features of the Suitability Assessment of PLES in the YRB

The exploratory spatial data analysis method was used to measure the spatial agglomeration degree of PLES functions with ArcGIS 10.2. The findings revealed that the Moran’s I of ecological function importance was 0.2321, 0.2682 and 0.2042 in 2010, 2015 and 2020, respectively. The Moran’s I of production function suitability was 0.2824, 0.4411 and 0.2824 in 2010, 2015 and 2020, respectively. The Moran′s I of life function suitability was 0.1378, 0.1083 and 0.1201 in 2010, 2015 and 2020, respectively. The P value of the suitability of “ecological-production-living” in 2010, 2015 and 2020 was less than 0.05, and the Z value exceeds the critical value, so the global Moran’s I passes the test, indicating that the function of PLES has positive spatial autocorrelation. The agglomeration characteristics of ecological importance and production suitability are strong, while the agglomeration characteristics of life function suitability are relatively weak (Table 3).

**Figure 9:**
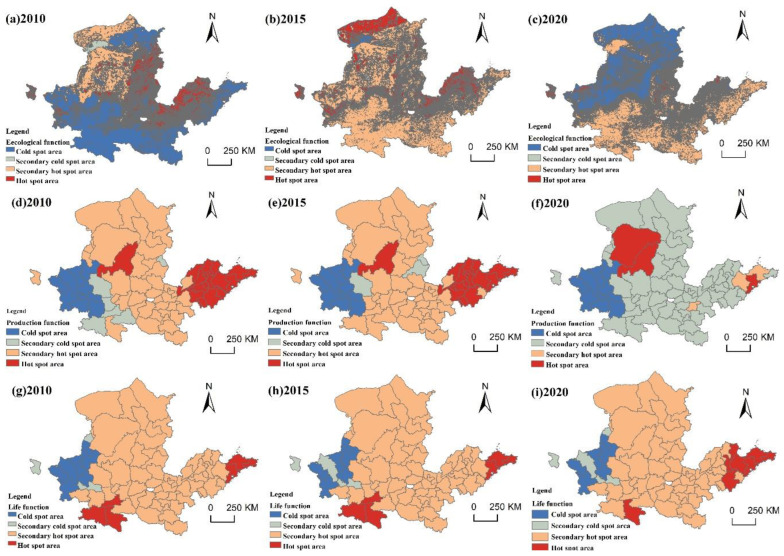
Agglomeration characteristics of the suitability of PLES.

Due to the possible defects of global autocorrelation in terms of local instability, the Gi* index of urban functional suitability in the YRB was further divided into four types of areas, specifically including hot spots, second hot spots, cold spots, and second cold spots, using the natural breakpoint method through local spatial autocorrelation analysis (Figure 9). The hot spots of ecological importance in 2010 were mainly concentrated in Yantai, Weifang, Zibo, Jinan, Yangquan, Luliang, Xinzhou, Shuozhou, Datong, Yulin, Yan’an, Lanzhou and Xining. In 2015, although the Shandong Peninsula urban agglomeration was still a hot spot, it moved to the west, with the overall cold spot rising to the second cold spots or second hot spots, and some of the second hot spots rising to hot spots. In 2020, the northwest and southeast regions showed significant differences. The northwest region was dominated by cold spots, and the second cold spots, the second hot spots and the hot spots were scattered, while the southeast region was dominated by second hot spots, and hot spots and second cold spots were scattered. In 2010, the hot spots of production suitability were mainly distributed in Jinan, Qingdao, Yantai, Jining, Heze, Puyang, Yulin and other cities. The second hot spots are mainly concentrated in Zhengzhou, Kaifeng, Luoyang, Nanyang, Yanan, Tongchuan, Weinan, Taiyuan, Datong, Luliang, Jincheng, Huhehaote, Ulanqab, Baotou, Erdos, Xining and other cities. The second cold spots are distributed in Yangquan, Xian, Shangluo, Hanzhong, Qingyang and Xianyang. Most of the cold spots are located in Lanzhou, Yinchuan, Dingxi, Baiyin, Zhongwei and other cities; there was no significant difference between 2015 and 2010. The hot spots in eastern cities decreased to second hot spots, and some cities in the southwest increased to second hot spots, while the cold spots remained unchanged. In 2020, all second hot spots were reduced to second cold spots, most of the hot spots were reduced to second hot spots or second cold spots, the cold spots remained unchanged, and Erdos was upgraded to the hot spots. The hot spots of life suitability in 2010 were mainly distributed in Qingdao, Yantai, Weihai, Xian, Hanzhong and Ankang. The second cold spots were distributed in Xining, Pingliang and Shizuishan. The cold spots were mainly distributed in Lanzhou, Guyuan, Tianshui and other cities. In 2015, the distribution was roughly the same as that in 2010, with only Baiyin city rising to the second cold spots. In 2020, there was still no significant change. Some cities in Shandong Peninsula became hot spots, while Hanzhong and Ankang became second hot spots.

### 4.3. Factor Analysis of the Spatial Suitability Obstacle Degree of PLES in the YRB

**Figure 10:**
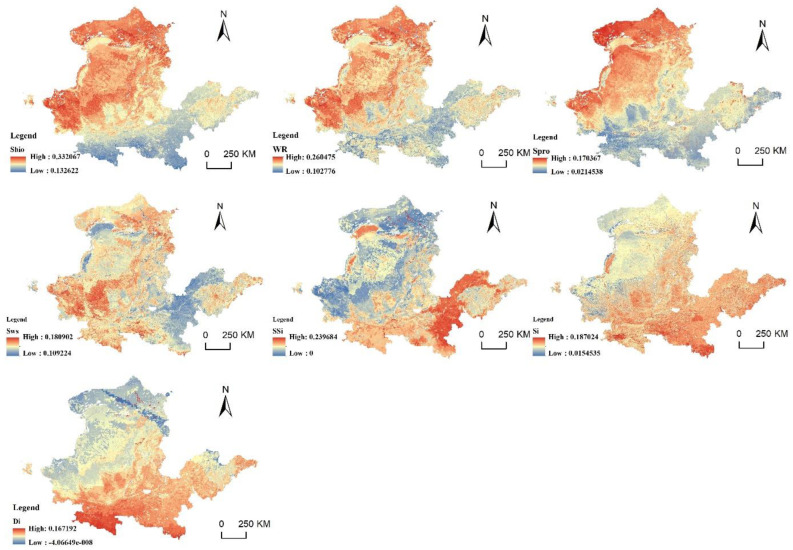
Ecological-importance barriers in 2010.

**Figure 11:**
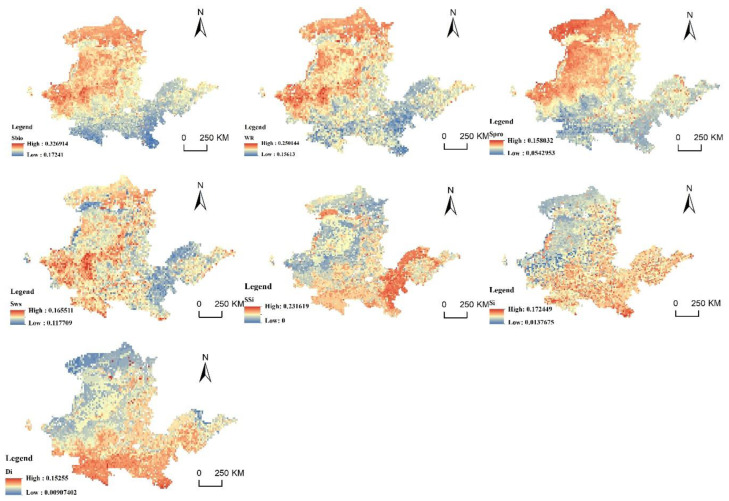
Ecological-importance barriers in 2015.

**Figure 12:**
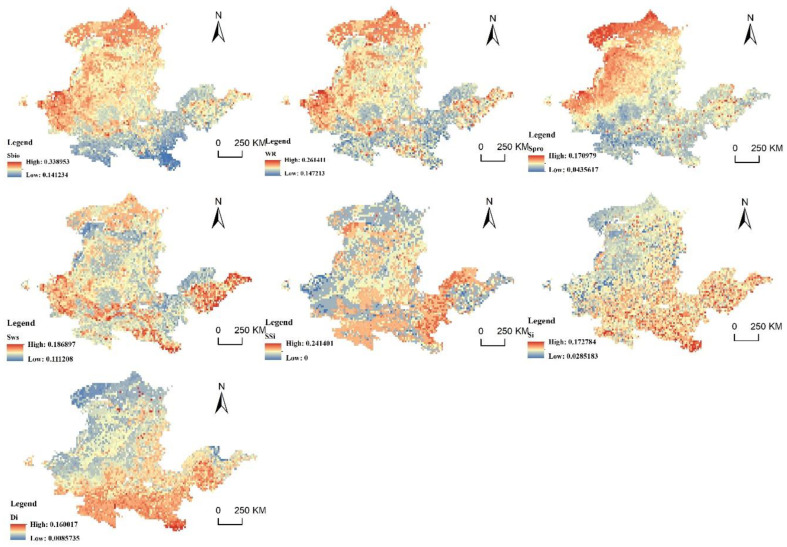
Ecological-importance barriers in 2020.

The obstacle degree model was utilized to identify some contributing aspects that influence the suitability of PLES in the YRB territorial space. The calculation results of the obstacle degree of a single indicator of regional ecological importance are shown in Figure 10, Figure 11 and Figure 12. In 2010, 2015 and 2020, biodiversity, the significance of water-source conservation and the significance of windbreaks and sand fixation functions were the primary impediments to the YRB’s ecological importance. Biodiversity, the significance of water conservation, the significance of soil and water conservation and the significance of windbreak and sand fixation have similar distribution patterns, which are essentially the importance level of ecosystem services, showing a higher obstacle degree in northwest direction and lower obstacle degree in southeast direction. As the northwest is the upper reaches of the YRB and the water-source conservation of YRB areas is in the upper reaches, the region’s ecosystem is more delicate. The sensitivity to soil and water loss, land desertification and rock desertification showed a low obstacle degree in the upper stream of the basin and a high obstacle degree in the middle–low stream of the basin. The rapid development of industrial enterprises in the middle and lower reaches has aggravated the damage to the ecological environment, resulting in environmental pollution and making the ecosystem more sensitive.

In 2010, the average obstacle degrees of biodiversity, significance of water-source conservation, and significance of windbreaks and sand fixation were 26.28%, 20.83% and 14.29%, respectively. In 2015, the average obstacle degrees were 26.53%, 25.01% and 14.31%, respectively. The average obstacle degree in 2020 was 25.84%, 20.29% and 13.94%, respectively, showing a rough trend of decreasing obstacle degree. The obstacle degree of land-desertification sensitivity was 7.82% in 2010, 8.01% in 2015 and 8.2% in 2020, showing a trend of a gradual increase. In the context of global efforts to promote biodiversity conservation, China has achieved remarkable results after decades of efforts. Chinese efforts to protect the environment and the ecological system have improved collaboration, strengthened the protection of grassland and wetland areas in water-conservation ecological function zones, and promoted the improvement of ecological and environmental quality of the YRB. The YRB’s biological surroundings have shown remarkable improvement, but they still face some problems at present. Since 2010, China’s weather and climate have been unusual: precipitation has increased; high temperatures, drought and waterlogging disasters have been connected; cold and hot space and time alternations have been obvious; extreme weather and climate events have occurred; and heavy rainfall has resulted in multiple river-basin floods and frequent geological disasters. Thus, the land-desertification problem in the YRB continues to be serious.

The obstacle factors of PLES suitability of each city in the study area in 2010, 2015 and 2020 were calculated using Equations (3) and (4), and the top three obstacle factors were listed, as shown in Table A1 and Table A2. The top three obstacle factors to production suitability add up to a total of degrees above 76%, demonstrating that these variables have the greatest impact on all fundamental indicators. The output of industrial added value per unit of industrial land (228 times, average obstacle degree 40.14%), number of enterprises above designated size (226 times, average obstacle degree 40.14%), average obstacle degree (29.47%) and GDP per capita (212 times, average obstacle degree 15.88%) were the dominant factors affecting the suitability of production in the study area. The output of industrial added value per unit of industrial land, the number of industrial enterprises above a designated size and per capita GDP are conducive to improving production capacity and efficiency, providing employment opportunities, and improving the capacity of social security. Income levels affect consumption ability, and then increase production. At the same time, the improvement of residents’ income level creates life demands, promoting the development of the service sector and promoting the perfection of the production function and living function.

Among the primary impediment factors affecting the production suitability of cities along the YRB, the frequency of the output of industrial added value per unit of industrial land showed a decreasing trend (77 times in 2010, 76 times in 2015, and 75 times in 2020). The urbanization rate (once in 2010, three times in 2015 and five times in 2020) and the output value of secondary and tertiary industries per unit of construction land (0 times in 2010, 6 times in 2015 and 12 times in 2020) are gradually increasing. The pattern of economic development has also changed from extensive, low-quality and high-quantity to concentrate on the quality of development, continuous improvement of the economic structure, and fast growth of tertiary and secondary industries. The urbanization rate is conducive to guiding the flow of production components that are two-way, such as land, capital and labour, producing an agglomeration scale effect and resource-allocation wealth effect, and then promoting the transformation, development, enhancement and improvement of production capacity, industrial structure and employment structure, which can effectively improve the suitability of production.

Through the analysis of occurrence frequency and average obstacle degree, it is discovered that the most typical barrier factors affecting living suitability mainly include total water use per capita (223 times, average obstacle degree 32.47%), total sewage treatment per capita (220 times, average obstacle degree 19.29%), and per-capita residential land area (times 183, average obstacle degree 14.37%). First, the outstanding total water-resource utilization per capita is a barrier factor because the index levels of Xian, Jinan, Qingdao, Zhengzhou, and Yinchuan are significantly better than those of other cities. These cities have a higher level of economic development and need more water resources than other cities. With the expansion of ecological civilization construction, the urban demand for sewage treatment is increasing daily. Especially given the swift economic growth, the increase in sewage-treatment volume can satisfy urban inhabitants’ needs for a green lifestyle. The appearance of per-capita residential land area highlights that the cities along the YRB have a comparatively modest level of urbanization. Shandong and Henan, as provinces with large populations, still need to solve the outstanding problem of the man–land contradiction.

The interannual variation in the main obstacle variables was considered, and almost all the top three major obstacle variables of urban living suitability along the YRB in 2010 included the total water use per capita, total sewage treatment per capita, and per-capita residential land area. By 2020, the main barriers remained the same across cities. However, the frequency of total water use per capita and the total amount of sewage treatment per capita decreased, while the frequency of the number of medical practitioners per 1000 population increased rapidly. The country strengthened the conceptualisation of a water-saving and anti-pollution society, which strengthened the Chinese people’s understanding of water conservation and reduces water pollution. However, improving the quality of medical treatment is the main means to maintain the health of all people. The society’s requirement for life is not only to have enough food and clothing but also to seek a high-quality and healthy lifestyle.

## 5. Discussion

The ecological importance level of cities along the YRB has increased overall from 2010 to 2015 and established more obvious zoning in geographic space in 2020, mainly because China’s biodiversity conservation work is effective. However, because the YRB spans a large area, there are large differences in resources and the environment, resulting in the formation of obvious zones of ecological importance; from 2010 to 2020, the cities with higher production suitability are mainly distributed in the downstream, mainly because the Guanzhong-Tianshui region, the Hubao-Egyu region, the Lanzhou-Xining region, and the Central Plains Economic Zone have certain scientific and technological innovation capacities and industrial development potential. The optimized development zones, mainly in the Jiaodong Peninsula, the Yellow River Delta region and the provincial central cities in the basin, have strong comprehensive strength and high production suitability; the suitability of life as a whole continually improved from 2010 to 2020, and the higher suitability level is for some provincial capitals and their surrounding cities. The main reason is that these cities are at a higher level than other cities in terms of transportation and economy, and their infrastructure is relatively complete, which greatly improves the suitability for living.

In this paper, the technical framework of PLES delineation in the carrying-capacity evaluation of resources and the environment and suitability evaluation of the spatial planning of the YRB was constructed using precise and simple evaluation indexes and quantitative and modelling evaluation methods. The method has the following advantages: (1) In the evaluation method, the traditional method of the carrying-capacity evaluation of resources and environment and suitability evaluation is optimized and quantitative models such as the entropy weight method are introduced to carry out the comprehensive evaluation of multiple elements, which makes the evaluation results more scientific. (2) In the traditional carrying-capacity evaluation of resources and the environment and the suitability evaluation system of ecology, agriculture and towns, a multi-level evaluation system based on the concept of PLE is established, and the relatively defined concept of PLE is implemented into this process through the screening of evaluation indexes. (3) In terms of evaluation indicators, the number of socio-economic indicators are increased, this improves the authenticity and scientificity of evaluation results, and plays a better supporting role in serving the preparation of territorial spatial planning. Limitations are, mainly: (1) the model evaluation method is relatively accurate, but the data processing and quality requirements are high due to the vector or raster data of soil and water resources, environment, temperature and rainfall included in the indicators measuring ecological importance. (2) Some data are not easy to obtain and the accuracy of the data is not high, which makes the evaluation results of average accuracy, and we will try to obtain more accurate data in the future.

## 6. Conclusions

(1) From 2010 to 2015, overall, the ecological importance of the cities along the YRB rose, but the magnitude was small. In 2020, cities near the YRB experienced a significant change in their ecological importance level, forming a relatively obvious partition in geographical space. From 2010 to 2020, most cities with higher production suitability were spread downstream, and the maximum number of cities with higher production suitability were present in 2015. From 2010 to 2020, the overall living suitability continued to improve, and the higher level of suitability was in some provincial capitals and surrounding cities. (2) Positive spatial autocorrelation of PLES. The agglomeration characteristics of ecological importance and production suitability are strong, while the agglomeration characteristics of life-function suitability are relatively weak. The distribution areas of cold hot spots of the PLES function overlap in space, to some extent. Most of the cold spots are located in the YRB’s higher reaches, and most of the hot spots or second hot spots were spread in the middle and lower reaches in the YRB. (3) From 2010 to 2020, biodiversity, water conservation and windbreak and sand fixation were the primary impediments to the ecological relevance of the YRB. The output of industrial added value per unit of industrial land, the number of industrial enterprises and GDP per capita were the dominant factors affecting the production suitability of the study area. Among the top three major obstacles to urban-living suitability along the YRB, almost all cities experience the per-capita total water-resource utilization, total sewage treatment per capita, and per-capita residential land area. However, the first three barriers are becoming increasingly less obstructive. Based on the above findings, the following recommendations are made:

(1) By defining key functional domains, the balanced growth of various functions can be achieved. With the basic idea of ecological civilization running through the whole process, all departments, provinces, and autonomous areas in the YRB must break through administrative restrictions, plan spatial distribution from a strategic level, optimize spatial structure, and achieve spatial balance.

According to the different regional features of the different watershed segments, the protection of the main flow corridors and migration channels should be coordinated in all regions. National parks will be established in the Yellow River Delta and other priority areas for biodiversity conservation. In the YRB’s middle and lower reaches, new nature reserves, ecological protection red lines or protected areas will be established, and the connectivity of existing nature reserves will be strengthened.

To implement water-source protection using watersheds, the upstream should closely follow the functional orientation of water conservation and strictly follow the principles of sustainability and efficiency to increase water-resource efficiency. Water and soil conservation should be placed in a prominent position in the middle reaches, and high-energy and high-pollution enterprises should be eliminated. Downstream the river ecosystem’s healthy and green development should be enhanced and the sensitivity of the ecological environment reduced.

Major projects should be implemented concerning important-ecosystem protection and restoration, establishing ecological space layouts, matching production and living constantly to improve the quality of sandy plantations. Continuing the implementation of major projects, these should focus on regional ecological protection and restoration, accelerating land desertification, salinization, and vegetation restoration in mining management, and strive to build a new development pattern for combating desertification.

(2) Upstream key ecological-function areas and main agricultural product-producing areas should focus on environmental protection, develop an ecological economy and industries that can be sustained by resources and the environment according to local conditions, and should link their own characteristics and advantages with the overall industrial layout of the watershed according to the layout of the main functional areas upstream, thus improving the level of upstream production suitability.

In view of the different stages of economic development, differentiated development strategies should be adopted. Industrialization in the eastern coastal area started early, and most cities are at an intermediate and advanced degree of industrialization. However, the central and western regions have been responsible for the country’s main agricultural production, and the industrial base is weak. Therefore, we should pay attention to the interaction between regions, promote the coordinated development between regions, and stimulate positive spatial interaction. According to the original industrial characteristics and their own resource advantages, each region should reasonably plan its industrial layout to form a reasonable division of labour and specialization among regions and to form an industrial cooperation mechanism among regions. The eastern region has a relatively higher suitability level and can provide more funds for the central and western regions, advanced technology and high-level talented people. The central and western regions are rich in resources and can deliver more clean energy to the eastern region. The overall suitability level of the YRB will be improved under the joint action of the west and east regions. This should actively guide the reasonable distribution of the population in cities and surrounding areas and actively develop industries in surrounding areas to promote industrial transfer in core areas.

The whole area should promote the transformation of the original resource-based industry into the direction of being fine, high-end and clustered, and promote the integration of manufacturing and service industries; large enterprises should be encouraged as the leaders of industrial development, the direction of industrial development should be clarified, and a scale industrial system formed with small- and medium-sized businesses; To strengthen the planning of the clustering of SMEs in the industrial sector, an industrial cluster system should be formed with a reasonable layout, industrial coordination, complete categories and international competitive advantages.

(3) The distribution and control of water resources in the upper reaches of the YRB, where there are few water resources, should be performed in accordance with the overall interests of the basin. The overall notion of “reducing agricultural water use, saving water for life, improving ecological water use and ensuring industrial water use” should be adhered to and the limited and scarce water resources exploited. In the middle and lower sections of the YRB, where there are plenty of water resources, within an appropriate range of the resource and ecosystem carrying capacity, water resources should be produced and used. The development philosophy of determining city, land, people and production should be adhered to using water, by refining the total water-use control index in the basin, by improving the water-resources control index system of YRB, and promoting the pattern of allocating water resources. This will continuously strengthen the control of the minimum flow required to maintain the river ecology and environment and implement the unified water-resources management of the main and tributaries of the YRB. To carry out pollution prevention and control, water conservation and control should be comprehensively implemented.

In the future, the government should prioritize raising the standard of cheap homes, to achieve a balance between employment and housing, and achieve the goal of “little but comfortable” to ensure the security, peace and comfort of inhabitants. Although the number of medical practitioners per 1,000 population in China is on the rise, there are obvious regional differences. Therefore, to reduce geographical variations in the availability of medical professionals, attention should be paid to the allocation of doctors in remote areas and to improving medical services in remote areas and mountain villages. In recent years, the demand gap of doctors in certain places has been eased to some extent, but it is a long way from the intended goal, which makes it challenging to meet the need, and the supply–demand disparity will continue for a very long time. In the future, it is necessary to implement the Opinions, strengthen the education of general-practice personnel, and actually succeed in the aim of “full coverage, filling up the weak points, improving the level and ensuring the quality”.

## Figures and Tables

**Table 1:** Resource and environment carrying-capacity evaluation index system.

Rule Layer	Factor Layer	Index Layer
Ecological importance	Ecosystem service function	Functional importance of biodiversity maintenance (Sbio)
		Significance of water conservation function (WR)
		Significance of soil and water conservation function (Spro)
		Significance of windbreak and sand fixation function (Sws)
	Ecological sensitivity	Water and soil loss sensitivity (SSi)
		Sensitivity of rocky desertification (Si)
		Sensitivity to desertification (Di)
Production carrying capacity	Carrying capacity of land resources	Proportion of industrial land (OV)
		Output of industrial added value per unit of industrial land (SD)
		Unit construction land output value of secondary and tertiary industries (SE)
	Water resources carrying capacity	Wastewater output per unit of additional industrial value (PI)
		Water consumption of CNY ten thousand of industrial value added (IV)
	Carrying capacity of environmental resources	Industrial smoke and dust emission per unit of additional industrial value (WC)
		Sulfur dioxide emissions per unit of additional industrial value (WD)
	Socioeconomic carrying capacity	Urbanization rate (UR)
		Number of enterprises above designated size (NI)
		GDP per capita (PGDP)
Life carrying capacity	Carrying capacity of land resources	Road area per citizen (PR)
		Per capita residential land area (RL)
	Water resources carrying capacity	Total water use per capita (TW)
		Total sewage treatment per capita (ST)
	Carrying capacity of environmental resources	Per capita park green area (PG)
		Harmless disposal rate of household garbage (HP)
	Socioeconomic carrying capacity	Expenditure on science education (ES)
		Annual disposable Income of Urban (DI)
		Number of medical practitioners per 1000 population (NP)

**Table 2:** Discriminant matrix of ecological protection importance.

Primary Judgement on the Importance of Ecological Protection	Ecological Plaque Concentration				
	Top Level	Upper Middle Level	Intermediate Level	Lower Level	Lowest Lever
Top level	Top level	Top level	Top level	Top level	Upper middle level
Upper middle level	Top level	Upper middle level	Upper middle level	Intermediate level	Intermediate level
Intermediate level	Upper middle level	Intermediate level	Intermediate level	Lower	Lower

**Table 3:** Global autocorrelation coefficient of suitability of PLES.

Correlation Coefficient	Importance of Ecological			Suitability of Production			Suitability of Life		
year	2010	2015	2020	2010	2015	2020	2010	2015	2020
Moran’s I	0.2320	0.2682	0.2042	0.2824	0.4411	0.2824	0.1378	0.1083	0.12014
*p* value	0	0	0	0	0	0	0	0.0230	0.0129
Z score	578.31	323.03	887.77	5.55	8.48	5.55	2.82	2.27	2.29
Significance level	0.010	0.0100	0.0100	0.0100	0.0100	0.0100	0.0100	0.0500	0.0500

## Data Availability

The administrative areas, DEM, NPP (Net Primary Productivity), NDVI (Normalized Difference Vegetation Index), population density, spatial distribution of terrestrial ecosystem types and land use data, and meteorological data used in this study are in the Institute of Resources and Environmental Sciences of the Chinese Academy of Sciences and the National Center for Environmental Information (NCEI) of the National Oceanic and Atmospheric Administration (NOAA) of the United States as fair open data. The economic and social statistics presented in this study are publicly available in the China Statistical Yearbook, the China Urban Statistical Yearbook, the China Regional Economic Statistical Yearbook, the statistical yearbooks of relevant provinces and cities, and the statistical bulletin platform of national economic and social development.

## Appendix A

**Table A1:** Main obstacle elements and order of their degree of impact on the suitability of urban production in the YRB from 2010 to 2020.

Cities	Year	Obstacle Factors and the Order Obstacle Degree			Cities	Year	Obstacle Factors and the Order Obstacle Degree		
		1	2	3			1	2	3
Ankang	2010	SD(0.3237)	NI(0.315)	PGDP(0.1716)	Qingyang	2010	NI(0.3411)	SD(0.2725)	PGDP(0.1946)
	2015	NI(0.3404)	SD(0.2945)	PGDP(0.1846)		2015	SD(0.3547)	NI(0.3106)	PGDP(0.1783)
	2020	NI(0.3829)	SD(0.2246)	PGDP(0.1986)		2020	NI(0.3655)	SD(0.2821)	PGDP(0.2083)
Anyang	2010	SD(0.3913)	NI(0.2707)	PGDP(0.1526)	Rizhao	2010	SD(0.4006)	NI(0.273)	PGDP(0.1337)
	2015	SD(0.3983)	NI(0.2676)	PGDP(0.1609)		2015	SD(0.4085)	NI(0.285)	PGDP(0.1349)
	2020	SD(0.4021)	NI(0.3001)	PGDP(0.1587)		2020	SD(0.4116)	NI(0.2936)	PGDP(0.1232)
Bayinnaoer	2010	SD(0.3501)	NI(0.3285)	PGDP(0.1445)	Sanmenxia	2010	NI(0.3522)	SD(0.3202)	PGDP(0.1626)
	2015	SD(0.3912)	NI(0.3009)	PGDP(0.1351)		2015	NI(0.3596)	SD(0.2935)	PGDP(0.1679)
	2020	SD(0.4031)	NI(0.3048)	PGDP(0.1213)		2020	SD(0.4223)	NI(0.3257)	PGDP(0.1166)
Baiyin	2010	SD(0.3727)	NI(0.278)	PGDP(0.1614)	Shangluo	2010	SD(0.377)	NI(0.305)	PGDP(0.1622)
	2015	SD(0.3734)	NI(0.2771)	PGDP(0.161)		2015	NI(0.4433)	PGDP(0.2287)	SD(0.1703)
	2020	SD(0.415)	NI(0.3076)	PGDP(0.179)		2020	NI(0.4099)	SD(0.2575)	PGDP(0.1893)
Baotou	2010	SD(0.4553)	NI(0.3157)	SE(0.1078)	Shangqiu	2010	SD(0.3554)	NI(0.2879)	PGDP(0.1685)
	2015	SD(0.4567)	NI(0.3185)	SE(0.0873)		2015	NI(0.4339)	PGDP(0.2963)	UR(0.1412)
	2020	SD(0.4505)	NI(0.3124)	SE(0.1103)		2020	NI(0.4433)	PGDP(0.3265)	UR(0.1747)
Baoji	2010	SD(0.389)	NI(0.2851)	PGDP(0.1439)	Shizuishan	2010	SD(0.3566)	NI(0.3271)	PGDP(0.1373)
	2015	SD(0.3942)	NI(0.2968)	PGDP(0.1465)		2015	SD(0.4075)	NI(0.3041)	PGDP(0.128)
	2020	SD(0.4306)	NI(0.2974)	PGDP(0.1256)		2020	SD(0.4672)	NI(0.3403)	PGDP(0.1176)
Binzhou	2010	SD(0.4044)	NI(0.2704)	PGDP(0.1372)	Shuozhou	2010	SD(0.3676)	NI(0.3312)	PGDP(0.1399)
	2015	SD(0.4296)	NI(0.2503)	PGDP(0.1397)		2015	NI(0.4604)	PGDP(0.2033)	SD(0.1402)
	2020	SD(0.4482)	NI(0.2436)	PGDP(0.1281)		2020	NI(0.4342)	SD(0.2325)	PGDP(0.1565)
Datong	2010	SD(0.3845)	NI(0.2885)	PGDP(0.1442)	Taiyuan	2010	SD(0.4222)	NI(0.2959)	PGDP(0.1269)
	2015	SD(0.3883)	NI(0.2992)	PGDP(0.1483)		2015	SD(0.4234)	NI(0.2996)	PGDP(0.1288)
	2020	SD(0.4545)	NI(0.3252)	PGDP(0.1488)		2020	SD(0.4499)	NI(0.2969)	SE(0.1209)
Dezhou	2010	SD(0.4575)	PGDP(0.1748)	NI(0.168)	Taian	2010	SD(0.4133)	NI(0.2501)	PGDP(0.1462)
	2015	SD(0.4868)	PGDP(0.1717)	NI(0.1388)		2015	SD(0.4591)	NI(0.2235)	PGDP(0.1567)
	2020	SD(0.4551)	NI(0.2216)	PGDP(0.1366)		2020	SD(0.4432)	NI(0.2499)	PGDP(0.1371)
Dingxi	2010	SD(0.3693)	NI(0.2806)	PGDP(0.1615)	Tianshui	2010	SD(0.3726)	NI(0.277)	PGDP(0.1616)
	2015	SD(0.3737)	NI(0.2806)	PGDP(0.1633)		2015	SD(0.374)	NI(0.2816)	PGDP(0.1638)
	2020	SD(0.3751)	NI(0.2846)	PGDP(0.1649)		2020	SD(0.415)	NI(0.3104)	PGDP(0.1815)
Dongying	2010	SD(0.4542)	NI(0.3218)	SE(0.092)	Tongchuan	2010	SD(0.3928)	NI(0.2943)	PGDP(0.1434)
	2015	SD(0.482)	NI(0.3369)	SE(0.059)		2015	SD(0.3963)	NI(0.297)	PGDP(0.1397)
	2020	SD(0.4868)	NI(0.3158)	SE(0.0948)		2020	SD(0.4562)	NI(0.3386)	PGDP(0.136)
Ordos	2010	NI(0.4542)	SD(0.3336)	SE(0.1233)	Weihai	2010	SD(0.4594)	NI(0.245)	SE(0.1055)
	2015	NI(0.6508)	SD(0.1886)	UR(0.0645)		2015	SD(0.4811)	NI(0.2371)	SE(0.1044)
	2020	NI(0.6235)	SD(0.2306)	UR(0.0604)		2020	SD(0.4759)	NI(0.2713)	SE(0.1111)
Guyuan	2010	SD(0.3723)	NI(0.283)	PGDP(0.1521)	Weifang	2010	SD(0.5438)	PGDP(0.1911)	SE(0.106)
	2015	SD(0.3679)	NI(0.2878)	PGDP(0.1509)		2015	SD(0.5442)	PGDP(0.1916)	NI(0.0782)
	2020	SD(0.4192)	NI(0.3295)	PGDP(0.1541)		2020	SD(0.6204)	PGDP(0.1804)	UR(0.0759)
Hanzhong City	2010	SD(0.3849)	NI(0.2939)	PGDP(0.1595)	Weinan	2010	SD(0.3726)	NI(0.2804)	PGDP(0.1537)
	2015	SD(0.3782)	NI(0.3092)	PGDP(0.1637)		2015	SD(0.3552)	NI(0.3097)	PGDP(0.1671)
	2020	SD(0.4249)	NI(0.2992)	PGDP(0.1459)		2020	SD(0.3917)	NI(0.3009)	PGDP(0.1519)
Heze	2010	SD(0.4121)	NI(0.2101)	PGDP(0.1688)	Wuhai	2010	SD(0.4341)	NI(0.3297)	SE(0.1217)
	2015	SD(0.473)	PGDP(0.1922)	NI(0.1391)		2015	NI(0.4121)	SD(0.3249)	PGDP(0.1237)
	2020	SD(0.4504)	NI(0.1926)	PGDP(0.1641)		2020	NI(0.3835)	SD(0.3662)	SE(0.1549)
Hebi	2010	SD(0.3933)	NI(0.2799)	PGDP(0.1403)	Wulanchabu	2010	NI(0.3265)	SD(0.3231)	PGDP(0.1619)
	2015	SD(0.4003)	NI(0.283)	PGDP(0.141)		2015	NI(0.3411)	SD(0.3033)	PGDP(0.1646)
	2020	SD(0.4123)	NI(0.298)	PGDP(0.1167)		2020	SD(0.355)	NI(0.3243)	PGDP(0.1395)
Hohhot,	2010	SD(0.4222)	NI(0.3152)	SE(0.1072)	Wuzhong	2010	SD(0.3554)	NI(0.2983)	PGDP(0.1541)
	2015	SD(0.4208)	NI(0.3248)	SE(0.1015)		2015	SD(0.3828)	NI(0.2845)	PGDP(0.1493)
	2020	SD(0.419)	NI(0.3157)	SE(0.1239)		2020	SD(0.3944)	NI(0.2901)	PGDP(0.1309)
Jinan	2010	SD(0.4633)	NI(0.2355)	PGDP(0.1202)	Wuwei	2010	SD(0.3716)	NI(0.2786)	PGDP(0.1624)
	2015	SD(0.4759)	NI(0.2221)	PGDP(0.1263)		2015	NI(0.3248)	SD(0.287)	PGDP(0.1916)
	2020	SD(0.5532)	NI(0.187)	SE(0.1098)		2020	NI(0.3107)	SD(0.3053)	PGDP(0.1812)
Jining	2010	SD(0.4894)	PGDP(0.1783)	NI(0.1268)	Xi’an	2010	SD(0.4328)	NI(0.27)	PGDP(0.1374)
	2015	SD(0.472)	NI(0.1743)	PGDP(0.1648)		2015	SD(0.4403)	NI(0.2661)	PGDP(0.1416)
	2020	SD(0.4844)	NI(0.1929)	PGDP(0.1481)		2020	SD(0.4951)	NI(0.2214)	SE(0.1193)
Jiayuguan	2010	SD(0.3932)	NI(0.3076)	PGDP(0.1662)	Xining	2010	SD(0.3963)	NI(0.3017)	PGDP(0.1418)
	2015	SD(0.3926)	NI(0.3032)	PGDP(0.1674)		2015	SD(0.3634)	NI(0.3312)	PGDP(0.1515)
	2020	SD(0.4237)	NI(0.322)	PGDP(0.1756)		2020	SD(0.3929)	NI(0.3228)	PGDP(0.1285)
Jiaozuo	2010	SD(0.3968)	NI(0.2731)	PGDP(0.1432)	Xianyang	2010	SD(0.3942)	NI(0.2786)	PGDP(0.1503)
	2015	SD(0.4214)	NI(0.2617)	PGDP(0.1452)		2015	SD(0.4065)	NI(0.2805)	PGDP(0.1516)
	2020	SD(0.4401)	NI(0.2768)	PGDP(0.1272)		2020	SD(0.4329)	NI(0.2923)	PGDP(0.1347)
Jinchang	2010	SD(0.3782)	NI(0.2875)	PGDP(0.1599)	Xinzhou	2010	SD(0.3743)	NI(0.2935)	PGDP(0.1587)
	2015	SD(0.3811)	NI(0.2872)	PGDP(0.1623)		2015	SD(0.3746)	NI(0.2975)	PGDP(0.1619)
	2020	SD(0.426)	NI(0.3239)	PGDP(0.1794)		2020	NI(0.3792)	SD(0.2802)	PGDP(0.1878)
Jincheng	2010	SD(0.3696)	NI(0.3144)	PGDP(0.1441)	Xinxiang	2010	SD(0.4031)	NI(0.2515)	PGDP(0.1559)
	2015	SD(0.3412)	NI(0.3398)	PGDP(0.1574)		2015	SD(0.4158)	NI(0.247)	PGDP(0.1607)
	2020	SD(0.3859)	NI(0.3567)	PGDP(0.1372)		2020	SD(0.4545)	NI(0.2429)	PGDP(0.1592)
Jinzhong	2010	SD(0.3879)	NI(0.2874)	PGDP(0.1495)	Xinyang	2010	SD(0.4001)	NI(0.2527)	PGDP(0.1586)
	2015	SD(0.3891)	NI(0.2883)	PGDP(0.1552)		2015	SD(0.4118)	NI(0.2514)	PGDP(0.1657)
	2020	SD(0.4109)	NI(0.2616)	PGDP(0.1379)		2020	SD(0.4465)	NI(0.2477)	PGDP(0.1509)
Jiuquan	2010	SD(0.3707)	NI(0.2924)	PGDP(0.1703)	Xuchang	2010	SD(0.3911)	NI(0.2701)	PGDP(0.1542)
	2015	SD(0.3716)	NI(0.294)	PGDP(0.1736)		2015	SD(0.4027)	NI(0.2667)	PGDP(0.1718)
	2020	SD(0.3683)	NI(0.294)	PGDP(0.1709)		2020	SD(0.4663)	NI(0.2698)	PGDP(0.1322)
Kaifeng	2010	SD(0.3997)	NI(0.2475)	PGDP(0.1522)	Yantai	2010	SD(0.4969)	NI(0.1615)	PGDP(0.1263)
	2015	SD(0.4124)	NI(0.2382)	PGDP(0.1553)		2015	SD(0.5015)	NI(0.1833)	PGDP(0.127)
	2020	SD(0.4321)	NI(0.2485)	PGDP(0.1378)		2020	SD(0.5484)	NI(0.191)	SE(0.0923)
Laiwu	2010	SD(0.3891)	NI(0.2986)	PGDP(0.1279)	Yan’An	2010	NI(0.5883)	PGDP(0.2403)	UR(0.1058)
	2015	SD(0.4021)	NI(0.2725)	PGDP(0.1356)		2015	NI(0.5715)	PGDP(0.2496)	UR(0.0933)
	2020	SD(0.4458)	PGDP(0.197)	NI(0.156)		2020	NI(0.6159)	PGDP(0.2151)	UR(0.1074)
Lanzhou	2010	SD(0.3937)	NI(0.285)	PGDP(0.173)	Yangquan	2010	SD(0.3951)	NI(0.3019)	PGDP(0.1367)
	2015	SD(0.3975)	NI(0.2867)	PGDP(0.1714)		2015	SD(0.3976)	NI(0.3083)	PGDP(0.1413)
	2020	SD(0.4471)	NI(0.3152)	PGDP(0.19)		2020	SD(0.415)	NI(0.3123)	PGDP(0.1233)
Liaocheng	2010	SD(0.4453)	NI(0.2084)	PGDP(0.1635)	Yinchuan	2010	SD(0.41)	NI(0.3021)	PGDP(0.1316)
	2015	SD(0.4663)	PGDP(0.1829)	NI(0.1704)		2015	SD(0.4162)	NI(0.3021)	PGDP(0.127)
	2020	SD(0.4371)	NI(0.2124)	PGDP(0.1464)		2020	SD(0.48)	NI(0.3516)	PGDP(0.1279)
Linfen	2010	NI(0.3387)	SD(0.3129)	PGDP(0.1781)	Yulin.	2010	NI(0.4438)	SD(0.256)	PGDP(0.1752)
	2015	NI(0.4101)	PGDP(0.2177)	SD(0.1698)		2015	NI(0.3851)	SD(0.3255)	PGDP(0.1641)
	2020	NI(0.4145)	PGDP(0.2076)	SD(0.2035)		2020	NI(0.6577)	PGDP(0.1536)	UR(0.146)
Linyi	2010	SD(0.474)	PGDP(0.1825)	SE(0.1144)	Yuncheng	2010	SD(0.3642)	NI(0.2923)	PGDP(0.163)
	2015	SD(0.5149)	PGDP(0.1981)	SE(0.1078)		2015	SD(0.3789)	NI(0.2972)	PGDP(0.1661)
	2020	SD(0.5631)	PGDP(0.185)	SE(0.0951)		2020	SD(0.3765)	NI(0.2963)	PGDP(0.1955)
Luoyang	2010	SD(0.4185)	NI(0.232)	PGDP(0.1439)	Zaozhuang	2010	SD(0.4115)	NI(0.2381)	PGDP(0.1451)
	2015	SD(0.4369)	NI(0.2227)	PGDP(0.1541)		2015	SD(0.4243)	NI(0.2457)	PGDP(0.1491)
	2020	SD(0.4901)	NI(0.2196)	PGDP(0.1291)		2020	SD(0.41)	NI(0.2628)	PGDP(0.1339)
Luohe	2010	SD(0.3724)	NI(0.2907)	PGDP(0.1504)	Zhangye	2010	SD(0.3741)	NI(0.2784)	PGDP(0.1615)
	2015	SD(0.3649)	NI(0.2997)	PGDP(0.1592)		2015	SD(0.366)	NI(0.2873)	PGDP(0.1681)
	2020	SD(0.4108)	NI(0.316)	PGDP(0.1249)		2020	SD(0.3847)	NI(0.2926)	PGDP(0.17)
Luliang	2010	SD(0.369)	NI(0.3406)	PGDP(0.1807)	Changzhi	2010	SD(0.3757)	NI(0.2991)	PGDP(0.1457)
	2015	SD(0.3674)	NI(0.3356)	PGDP(0.1858)		2015	SD(0.3846)	NI(0.2966)	PGDP(0.1492)
	2020	SD(0.4067)	NI(0.3246)	PGDP(0.1572)		2020	SD(0.4143)	NI(0.3024)	PGDP(0.1322)
Nanyang	2010	SD(0.3958)	NI(0.2518)	PGDP(0.1667)	Zhengzhou	2010	SD(0.4547)	NI(0.2064)	PGDP(0.1482)
	2015	SD(0.4415)	NI(0.1912)	PGDP(0.1747)		2015	SD(0.4695)	NI(0.1851)	PGDP(0.1669)
	2020	SD(0.4524)	NI(0.2277)	PGDP(0.1577)		2020	SD(0.544)	NI(0.1908)	PGDP(0.1095)
Pingdingshan	2010	SD(0.3888)	NI(0.2744)	PGDP(0.1525)	Zhongwei	2010	SD(0.3697)	NI(0.2848)	PGDP(0.1456)
	2015	SD(0.3985)	NI(0.2756)	PGDP(0.158)		2015	SD(0.3217)	NI(0.3183)	PGDP(0.1605)
	2020	SD(0.4285)	NI(0.2944)	PGDP(0.1512)		2020	NI(0.3295)	SD(0.3206)	PGDP(0.146)
Pingliang	2010	SD(0.365)	NI(0.2831)	PGDP(0.1626)	Zhoukou	2010	SD(0.3441)	NI(0.2914)	PGDP(0.1843)
	2015	SD(0.3687)	NI(0.282)	PGDP(0.1629)		2015	SD(0.3328)	NI(0.3057)	PGDP(0.2094)
	2020	SD(0.4087)	NI(0.3197)	PGDP(0.1826)		2020	SD(0.4047)	NI(0.268)	PGDP(0.1879)
Puyang	2010	SD(0.3543)	NI(0.2995)	PGDP(0.1639)	Zhumadian	2010	SD(0.4002)	NI(0.2423)	PGDP(0.1664)
	2015	SD(0.3612)	NI(0.2922)	PGDP(0.1719)		2015	SD(0.4168)	NI(0.2324)	PGDP(0.172)
	2020	SD(0.3826)	NI(0.3142)	PGDP(0.1562)		2020	SD(0.4232)	NI(0.2796)	PGDP(0.1509)
Qingdao	2010	SD(0.6215)	PGDP(0.1504)	SE(0.1195)	Zibo	2010	SD(0.4878)	NI(0.1549)	PGDP(0.1181)
	2015	SD(0.6424)	PGDP(0.1453)	SE(0.1168)		2015	SD(0.5071)	NI(0.1577)	PGDP(0.1313)
	2020	SD(0.7071)	SE(0.1335)	PGDP(0.0792)	cities	2020	SD(0.4832)	NI(0.206)	SE(0.1342)

**Table A2:** Main obstacle elements and order of their degree of impact on the suitability of urban living in the YRB from 2010 to 2020.

Cities	Year	Obstacle Factors and the Order Obstacle Degree			Cities	Year	Obstacle Factors and the Order Obstacle Degree		
		1	2	3			1	2	3
Ankang	2010	ST(0.2856)	RL(0.1842)	NP(0.1641)	Qingyang	2010	TW(0.2884)	ST(0.1855)	RL(0.1434)
	2015	ST(0.2764)	RL(0.1958)	NP(0.1486)		2015	TW(0.2877)	ST(0.1848)	RL(0.143)
	2020	ST(0.2482)	RL(0.1915)	NP(0.1546)		2020	TW(0.2897)	ST(0.1858)	RL(0.1446)
Anyang	2010	TW(0.3195)	ST(0.1778)	RL(0.1473)	Rizhao	2010	TW(0.3344)	ST(0.1928)	RL(0.1378)
	2015	TW(0.3061)	ST(0.1842)	RL(0.1379)		2015	TW(0.3243)	ST(0.1866)	RL(0.1227)
	2020	TW(0.3094)	ST(0.1813)	RL(0.1429)		2020	TW(0.2942)	ST(0.1757)	NP(0.1267)
Bayinnaoer	2010	TW(0.3115)	ST(0.1935)	RL(0.1295)	Sanmenxia	2010	TW(0.2718)	ST(0.1969)	RL(0.1454)
	2015	TW(0.3148)	ST(0.195)	ES(0.1223)		2015	TW(0.2771)	ST(0.1914)	RL(0.1264)
	2020	ST(0.2216)	RL(0.1604)	ES(0.1513)		2020	TW(0.2838)	ST(0.1854)	RL(0.1181)
Baiyin	2010	TW(0.2974)	ST(0.1885)	RL(0.1451)	Shangluo	2010	ST(0.2313)	RL(0.1772)	TW(0.1666)
	2015	TW(0.2895)	ST(0.1831)	RL(0.1411)		2015	TW(0.2356)	ST(0.2007)	RL(0.1551)
	2020	TW(0.2921)	ST(0.1856)	RL(0.1426)		2020	TW(0.2095)	ST(0.193)	RL(0.1566)
Baotou	2010	TW(0.3776)	ST(0.1878)	NP(0.1238)	Shangqiu	2010	TW(0.2996)	ST(0.1875)	RL(0.1482)
	2015	TW(0.3642)	ST(0.1849)	NP(0.1257)		2015	TW(0.2923)	ST(0.1821)	RL(0.1465)
	2020	TW(0.3624)	ST(0.1563)	ES(0.1305)		2020	TW(0.291)	ST(0.1853)	RL(0.1493)
Baoji	2010	TW(0.2935)	ST(0.194)	RL(0.1523)	Shizuishan	2010	TW(0.3929)	ST(0.1711)	ES(0.1514)
	2015	TW(0.287)	ST(0.1877)	RL(0.1538)		2015	TW(0.3661)	ST(0.1883)	ES(0.1473)
	2020	TW(0.2975)	ST(0.1681)	RL(0.1425)		2020	TW(0.3723)	ST(0.1693)	ES(0.1498)
Binzhou	2010	TW(0.3284)	ST(0.1982)	RL(0.1427)	Shuozhou	2010	TW(0.3135)	ST(0.1882)	RL(0.142)
	2015	TW(0.3209)	ST(0.1912)	RL(0.1273)		2015	TW(0.3004)	ST(0.1887)	RL(0.1289)
	2020	TW(0.3293)	ST(0.1862)	RL(0.1323)		2020	TW(0.2925)	ST(0.1825)	RL(0.1356)
Datong	2010	TW(0.328)	ST(0.1864)	RL(0.1227)	Taiyuan	2010	TW(0.3725)	RL(0.1318)	ST(0.1258)
	2015	TW(0.3172)	ST(0.1839)	NP(0.1089)		2015	TW(0.3598)	ST(0.1421)	ES(0.1085)
	2020	TW(0.3183)	ST(0.1648)	ES(0.1174)		2020	TW(0.3714)	RL(0.1201)	NP(0.1163)
Dezhou	2010	TW(0.3113)	ST(0.1976)	RL(0.163)	Taian	2010	TW(0.3482)	ST(0.2087)	RL(0.1458)
	2015	TW(0.3178)	ST(0.1869)	RL(0.1356)		2015	TW(0.3248)	ST(0.1976)	RL(0.1316)
	2020	TW(0.3217)	ST(0.1772)	RL(0.1394)		2020	TW(0.3164)	ST(0.1964)	RL(0.1379)
Dingxi	2010	TW(0.2854)	ST(0.1835)	RL(0.1425)	Tianshui	2010	TW(0.288)	ST(0.1853)	RL(0.1438)
	2015	TW(0.285)	ST(0.1837)	RL(0.1426)		2015	TW(0.2854)	ST(0.1829)	RL(0.1421)
	2020	TW(0.2854)	ST(0.1855)	RL(0.144)		2020	TW(0.2859)	ST(0.1828)	RL(0.143)
Dongying	2010	TW(0.3938)	ST(0.1874)	NP(0.1374)	Tongchuan	2010	TW(0.3118)	ST(0.1964)	ES(0.1299)
	2015	TW(0.3728)	ST(0.1881)	NP(0.1367)		2015	TW(0.3045)	ST(0.1859)	ES(0.1234)
	2020	TW(0.3827)	ST(0.1433)	ES(0.1337)		2020	TW(0.317)	ST(0.1743)	ES(0.1304)
Ordos	2010	TW(0.3669)	ST(0.2658)	NP(0.1386)	Weihai	2010	TW(0.3656)	ST(0.22)	RL(0.1423)
	2015	TW(0.3442)	ST(0.2382)	RL(0.1363)		2015	TW(0.372)	ST(0.196)	NP(0.1312)
	2020	TW(0.3145)	ST(0.2354)	RL(0.156)		2020	TW(0.3634)	ST(0.2015)	NP(0.1326)
Guyuan	2010	TW(0.3103)	ST(0.1966)	RL(0.123)	Weifang	2010	TW(0.37)	ST(0.2251)	RL(0.1634)
	2015	TW(0.3042)	ST(0.1927)	ES(0.1142)		2015	TW(0.3496)	ST(0.2089)	RL(0.1477)
	2020	TW(0.3056)	ST(0.2009)	RL(0.137)		2020	TW(0.3395)	ST(0.1991)	RL(0.1573)
Hanzhong City	2010	ST(0.2638)	RL(0.2108)	NP(0.1555)	Weinan	2010	TW(0.3127)	ST(0.1991)	RL(0.1472)
	2015	ST(0.2213)	RL(0.1767)	TW(0.1384)		2015	TW(0.3003)	ST(0.188)	RL(0.1374)
	2020	ST(0.2375)	RL(0.1873)	NP(0.147)		2020	TW(0.2965)	ST(0.1818)	RL(0.1395)
Heze	2010	TW(0.3031)	ST(0.202)	RL(0.1481)	Wuhai	2010	TW(0.4099)	ES(0.157)	ST(0.1434)
	2015	TW(0.2996)	ST(0.1917)	RL(0.1409)		2015	TW(0.4246)	ES(0.168)	NP(0.1382)
	2020	TW(0.3103)	ST(0.1953)	RL(0.1359)		2020	TW(0.409)	ES(0.1762)	NP(0.1551)
Hebi	2010	TW(0.3159)	ST(0.1715)	RL(0.13)	Wulanchabu	2010	TW(0.3121)	ST(0.2021)	RL(0.1408)
	2015	TW(0.3093)	ST(0.179)	ES(0.1221)		2015	ST(0.2963)	RL(0.189)	NP(0.1742)
	2020	TW(0.3145)	ST(0.1653)	RL(0.1288)		2020	ST(0.2795)	RL(0.1876)	ES(0.173)
Hohhot,	2010	TW(0.3609)	ST(0.1821)	NP(0.124)	Wuzhong	2010	TW(0.3273)	ST(0.1771)	RL(0.1269)
	2015	TW(0.3612)	ST(0.1908)	ES(0.1268)		2015	TW(0.3172)	ST(0.1808)	ES(0.1207)
	2020	TW(0.3546)	ST(0.1618)	NP(0.1259)		2020	TW(0.3188)	ST(0.1712)	RL(0.1266)
Jinan	2010	TW(0.3989)	ST(0.1763)	RL(0.1374)	Wuwei	2010	TW(0.2855)	ST(0.1834)	RL(0.1421)
	2015	TW(0.3899)	ST(0.1852)	RL(0.1229)		2015	TW(0.2857)	ST(0.1843)	RL(0.1402)
	2020	TW(0.4062)	ST(0.1659)	NP(0.135)		2020	TW(0.2853)	ST(0.1846)	RL(0.1424)
Jining	2010	TW(0.3539)	ST(0.213)	RL(0.1667)	Xi’an	2010	TW(0.3674)	ST(0.1593)	RL(0.1547)
	2015	TW(0.3321)	ST(0.1976)	RL(0.144)		2015	TW(0.368)	ST(0.1497)	RL(0.1315)
	2020	TW(0.3308)	ST(0.1984)	RL(0.1476)		2020	TW(0.3816)	ST(0.1401)	NP(0.1369)
Jiayuguan	2010	TW(0.3166)	ST(0.1847)	RL(0.1393)	Xining	2010	TW(0.3092)	ST(0.176)	NP(0.1099)
	2015	TW(0.318)	ST(0.1886)	RL(0.1359)		2015	TW(0.3176)	ST(0.1687)	ES(0.1223)
	2020	TW(0.3171)	ST(0.2049)	RL(0.1494)		2020	TW(0.307)	RL(0.1335)	ST(0.133)
Jiaozuo	2010	TW(0.3251)	ST(0.1718)	RL(0.1286)	Xianyang	2010	TW(0.3714)	RL(0.1453)	ST(0.1205)
	2015	TW(0.3162)	ST(0.173)	ES(0.116)		2015	TW(0.3917)	ES(0.1347)	RL(0.1153)
	2020	TW(0.3101)	ST(0.1766)	RL(0.1262)		2020	TW(0.3803)	ES(0.1437)	RL(0.1305)
Jinchang	2010	TW(0.3115)	ST(0.1978)	RL(0.1538)	Xinzhou	2010	TW(0.2878)	ST(0.1905)	RL(0.1431)
	2015	TW(0.3003)	ST(0.1913)	RL(0.1468)		2015	TW(0.2939)	ST(0.1948)	RL(0.1435)
	2020	TW(0.3023)	ST(0.1917)	RL(0.1486)		2020	TW(0.2852)	ST(0.19)	RL(0.146)
Jincheng	2010	TW(0.3156)	ST(0.1983)	RL(0.1266)	Xinxiang	2010	TW(0.3262)	ST(0.169)	RL(0.133)
	2015	TW(0.3091)	ST(0.1917)	ES(0.1189)		2015	TW(0.3179)	ST(0.1688)	RL(0.1228)
	2020	TW(0.2983)	ST(0.1798)	RL(0.1361)		2020	TW(0.3267)	ST(0.1726)	RL(0.1182)
Jinzhong	2010	TW(0.3079)	ST(0.1956)	RL(0.1442)	Xinyang	2010	TW(0.2395)	ST(0.2181)	RL(0.1637)
	2015	TW(0.3074)	ST(0.1906)	RL(0.138)		2015	TW(0.2498)	ST(0.2043)	RL(0.1491)
	2020	TW(0.3137)	ST(0.1876)	NP(0.12)		2020	TW(0.2168)	ST(0.2133)	RL(0.1564)
Jiuquan	2010	TW(0.2869)	ST(0.1929)	RL(0.1479)	Xuchang	2010	TW(0.3055)	ST(0.1911)	RL(0.1431)
	2015	TW(0.2839)	ST(0.1886)	RL(0.1447)		2015	TW(0.2983)	ST(0.1873)	RL(0.1399)
	2020	TW(0.2822)	ST(0.191)	RL(0.1481)		2020	TW(0.3109)	ST(0.1921)	RL(0.1463)
Kaifeng	2010	TW(0.3021)	ST(0.1798)	RL(0.1302)	Yantai	2010	TW(0.393)	ST(0.214)	RL(0.127)
	2015	TW(0.3042)	ST(0.1763)	RL(0.1226)		2015	TW(0.3689)	ST(0.1924)	RL(0.1254)
	2020	TW(0.3098)	ST(0.1754)	RL(0.1322)		2020	TW(0.3796)	ST(0.1935)	NP(0.1244)
Laiwu	2010	TW(0.3589)	ST(0.1858)	ES(0.1361)	Yan’An	2010	TW(0.2997)	ST(0.1941)	RL(0.1458)
	2015	TW(0.3633)	ST(0.192)	ES(0.142)		2015	TW(0.2945)	ST(0.1898)	RL(0.1435)
	2020	TW(0.4062)	ST(0.1659)	NP(0.135)		2020	TW(0.2898)	ST(0.1842)	RL(0.1438)
Lanzhou	2010	TW(0.3047)	ST(0.188)	RL(0.1459)	Yangquan	2010	TW(0.3141)	ST(0.1772)	RL(0.1321)
	2015	TW(0.2995)	ST(0.1859)	RL(0.1428)		2015	TW(0.3034)	ST(0.1764)	RL(0.1268)
	2020	TW(0.3074)	ST(0.1893)	RL(0.1476)		2020	TW(0.2967)	ST(0.1754)	RL(0.1299)
Liaocheng	2010	TW(0.3182)	ST(0.2072)	RL(0.1608)	Yinchuan	2010	TW(0.4271)	ES(0.1457)	NP(0.1317)
	2015	TW(0.3072)	ST(0.188)	RL(0.1369)		2015	TW(0.4506)	ES(0.1663)	NP(0.1097)
	2020	TW(0.3052)	ST(0.1812)	RL(0.1364)		2020	TW(0.4938)	ES(0.1744)	NP(0.1114)
Linfen	2010	TW(0.3098)	ST(0.1947)	RL(0.1442)	Yulin.	2010	TW(0.2988)	ST(0.2223)	RL(0.1388)
	2015	TW(0.2987)	ST(0.1911)	RL(0.1335)		2015	TW(0.2793)	ST(0.205)	RL(0.1469)
	2020	TW(0.2975)	ST(0.186)	RL(0.1381)		2020	TW(0.2831)	ST(0.2)	RL(0.1497)
Linyi	2010	TW(0.3436)	ST(0.2076)	RL(0.1625)	Yuncheng	2010	TW(0.3154)	ST(0.2011)	RL(0.1479)
	2015	TW(0.329)	ST(0.1936)	RL(0.1467)		2015	TW(0.2976)	ST(0.1964)	RL(0.1382)
	2020	TW(0.3078)	ST(0.2057)	RL(0.1609)		2020	TW(0.2961)	ST(0.1843)	RL(0.1451)
Luoyang	2010	TW(0.3072)	ST(0.1868)	RL(0.1328)	Zaozhuang	2010	TW(0.3293)	ST(0.1899)	RL(0.1329)
	2015	TW(0.3204)	ST(0.1825)	RL(0.1228)		2015	TW(0.3224)	ST(0.1848)	NP(0.1202)
	2020	TW(0.3271)	ST(0.1832)	RL(0.1275)		2020	TW(0.3078)	ST(0.1876)	RL(0.1223)
Luohe	2010	TW(0.3128)	ST(0.1618)	RL(0.1378)	Zhangye	2010	TW(0.2592)	ST(0.1954)	RL(0.1483)
	2015	TW(0.3102)	ST(0.1647)	RL(0.1323)		2015	TW(0.2705)	ST(0.2019)	RL(0.1437)
	2020	TW(0.3059)	ST(0.165)	RL(0.1382)		2020	TW(0.276)	ST(0.1904)	RL(0.1419)
Luliang	2010	TW(0.3063)	ST(0.2038)	RL(0.1542)	Changzhi	2010	TW(0.3053)	ST(0.1887)	RL(0.1497)
	2015	TW(0.2867)	ST(0.1929)	RL(0.1438)		2015	TW(0.2953)	ST(0.185)	RL(0.1444)
	2020	TW(0.2882)	ST(0.1833)	RL(0.1439)		2020	TW(0.2977)	ST(0.1824)	RL(0.1438)
Nanyang	2010	TW(0.2517)	ST(0.2168)	RL(0.1635)	Zhengzhou	2010	TW(0.4891)	NP(0.1215)	PR(0.114)
	2015	TW(0.2994)	ST(0.1959)	RL(0.1438)		2015	TW(0.5085)	PR(0.1233)	NP(0.0964)
	2020	TW(0.2946)	ST(0.1961)	RL(0.1486)		2020	TW(0.622)	PR(0.1471)	DI(0.0799)
Pingdingshan	2010	TW(0.2927)	ST(0.1749)	RL(0.1452)	Zhongwei	2010	TW(0.296)	ST(0.1887)	RL(0.1437)
	2015	TW(0.3055)	ST(0.1765)	RL(0.1345)		2015	TW(0.2948)	ST(0.1886)	RL(0.1445)
	2020	TW(0.3015)	ST(0.1714)	RL(0.1431)		2020	TW(0.3007)	ST(0.1919)	RL(0.148)
Pingliang	2010	TW(0.2963)	ST(0.1902)	RL(0.1463)	Zhoukou	2010	ST(0.2704)	TW(0.2418)	RL(0.1641)
	2015	TW(0.2883)	ST(0.185)	RL(0.1424)		2015	TW(0.2936)	ST(0.2147)	RL(0.1467)
	2020	TW(0.2908)	ST(0.1872)	RL(0.1446)		2020	TW(0.2492)	ST(0.2012)	RL(0.1457)
Puyang	2010	TW(0.2998)	ST(0.1896)	RL(0.1456)	Zhumadian	2010	TW(0.2967)	ST(0.2009)	RL(0.157)
	2015	TW(0.3059)	ST(0.1809)	RL(0.1346)		2015	TW(0.295)	ST(0.1916)	RL(0.1478)
	2020	TW(0.3092)	ST(0.1773)	RL(0.138)		2020	TW(0.2891)	ST(0.1934)	RL(0.1508)
Qingdao	2010	TW(0.4264)	ST(0.1877)	RL(0.1647)	Zibo	2010	TW(0.3687)	ST(0.165)	NP(0.1411)
	2015	TW(0.4239)	ST(0.1944)	NP(0.1396)		2015	TW(0.3545)	ST(0.1819)	NP(0.1298)
	2020	TW(0.4176)	ST(0.1751)	RL(0.1463)	cities	2020	TW(0.33)	ST(0.1641)	NP(0.1258)

## References

[1] Zuo Q., Zhou Y., Wang L., Li Q., Liu J. (2022). Impacts of future land use changes on land use conflicts based on multiple scenarios in the central mountain region, China. Ecol. Indic..

[2] Zhou D., Xu J., Lin Z. (2017). Conflict or coordination? Assessing land use multi-functionalization using production-living-ecology analysis. Sci. Total Environ..

[3] Chen Y., Zhu M., Zhou Q., Qiao Y. (2021). Research on Spatiotemporal Differentiation and Influence Mechanism of Urban Resilience in China Based on MGWR Model. Int. J. Environ. Res. Public Health.

[4] Lovell S.T., DeSantis S., Nathan C.A., Olson M.B., Ernesto Méndez V., Kominami H.C., Erickson D.L., Morris K.S., Morris W.B. (2010). Integrating agroecology and landscape multifunctionality in Vermont: An evolving framework to evaluate the design of agroecosystems. Agric. Syst..

[5] Kienast F., Bolliger J., Potschin M., de Groot R.S., Verburg P.H., Heller I., Wascher D., Haines-Young R. (2009). Assessing Landscape Functions with Broad-Scale Environmental Data: Insights Gained from a Prototype Development for Europe. Environ. Manag..

[6] Chen Y., Zhu M.K. (2022). Spatiotemporal Evolution and Driving Mechanism of “Production-Living-Ecology” Functions in China: A Case of Both Sides of Hu Line. Int. J. Environ. Res. Public Health.

[7] Li G.D., Fang C.L. (2016). Quantitative function identification and analysis of urban ecological-production-living spaces. Acta Geogr. Sin..

[8] Tao Y., Wang Q. (2021). Quantitative Recognition and Characteristic Analysis of Production-Living-Ecological Space Evolution for Five Resource-Based Cities: Zululand, Xuzhou, Lota, Surf Coast and Ruhr. Remote Sens..

[9] Zhang F., Tiyip T., Feng Z.D., Kung H.T., Johnson V.C., Ding J.L., Tashpolat N., Sawut M., Gui D.W. (2015). Spatio-Temporal Patterns of Land Use/Cover Changes over the Past 20 Years in the Middle Reaches of the Tarim River, Xinjiang, China. Land Degrad. Dev..

[10] Duan Y., Wang H., Huang A., Xu Y., Lu L., Ji Z. (2021). Identification and spatial-temporal evolution of rural “production-living-ecological” space from the perspective of villagers’ behavior—A case study of Ertai Town, Zhangjiakou City. Land Use Policy.

[11] Chen Y., Su X.Y., Wang X.K. (2022). Spatial Transformation Characteristics and Conflict Measurement of Production-Living-Ecology: Evidence from Urban Agglomeration of China. Int. J. Environ. Res. Public Health.

[12] Jiang S., Meng J., Zhu L., Cheng H. (2021). Spatial-temporal pattern of land use conflict in China and its multilevel driving mechanisms. Sci. Total Environ..

[13] Zhao L., Peng Z.-R. (2012). LandSys: An agent-based Cellular Automata model of land use change developed for transportation analysis. J. Transp. Geogr..

[14] Zou L., Liu Y., Wang J., Yang Y., Wang Y. (2019). Land use conflict identification and sustainable development scenario simulation on China’s southeast coast. J. Clean. Prod..

[15] Gong J., Liu Y., Chen W. (2012). Land suitability evaluation for development using a matter-element model: A case study in Zengcheng, Guangzhou, China. Land Use Policy.

[16] Jiang S., Meng J., Zhu L. (2020). Spatial and temporal analyses of potential land use conflict under the constraints of water resources in the middle reaches of the Heihe River. Land Use Policy.

[17] Xiao P.N., Xu J., Zhao C. (2022). Conflict Identification and Zoning Optimization of “Production-Living-Ecological” Space. Int. J. Environ. Res. Public Health.

[18] Han J., Hayashi Y., Cao X., Imura H. (2009). Application of an integrated system dynamics and cellular automata model for urban growth assessment: A case study of Shanghai, China. Landsc. Urban Plan..

[19] Arora A., Pandey M., Mishra V.N., Kumar R., Rai P.K., Costache R., Punia M., Di L. (2021). Comparative evaluation of geospatial scenario-based land change simulation models using landscape metrics. Ecol. Indic..

[20] Dezhkam S., Jabbarian A.B., Darvishsefat A.A., Sakieh Y. (2017). Performance evaluation of land change simulation models using landscape metrics. Geocarto Int..

[21] Yao H.R., Zheng D., Wu S.H. (2002). Optimum allocation of land and water in the typical sand regions around Beijing: A case study in Huailai County. Geogr. Res..

[22] Huang D., Huang J., Liu T. (2019). Delimiting urban growth boundaries using the CLUE-S model with village administrative boundaries. Land Use Policy.

[23] Talukdar S., Singha P., Mahato S., Shahfahad, Pal S., Liou Y.A., Rahman A. (2020). Land-Use Land-Cover Classification by Machine Learning Classifiers for Satellite Observations—A Review. Remote Sens..

[24] Chen G.Z., Li X., Liu X.P. (2022). Global land projection based on plant functional types with a 1-km resolution under socio-climatic scenarios. Sci. Data.

[25] Lin J., He P., Yang L., He X., Lu S., Liu D. (2022). Predicting future urban waterlogging-prone areas by coupling the maximum entropy and FLUS model. Sustain. Cities Soc..

[26] Baskent E.Z. (2021). Assessment and improvement strategies of sustainable land management (SLM) planning initiative in Turkey. Sci. Total Environ..

[27] Ustaoglu E., Aydınoglu A.C. (2020). Suitability evaluation of urban construction land in Pendik district of Istanbul, Turkey. Land Use Policy.

[28] Kaur H., Garg P. (2019). Urban sustainability assessment tools: A review. J. Clean. Prod..

[29] Hsu W.L., Shen X., Xu H., Zhang C., Liu H.L., Shiau Y.C. (2021). Integrated Evaluations of Resource and Environment Carrying Capacity of the Huaihe River Ecological and Economic Belt in China. Land.

[30] Zhang H., Wang Z., Liu J., Chai J., Wei C. (2019). Selection of targeted poverty alleviation policies from the perspective of land resources-environmental carrying capacity. J. Rural. Stud..

[31] Malczewski J. (2004). GIS-based land-use suitability analysis: A critical overview. Prog. Plann..

[32] Pourebrahim S., Hadipour M., Mokhtar M.B. (2011). Integration of spatial suitability analysis for land use planning in coastal areas; case of Kuala Langat District, Selangor, Malaysia. Landsc. Urban Plan..

[33] Liu Y., Fang F., Li Y. (2014). Key issues of land use in China and implications for policy making. Land Use Policy.

[34] Yang F., Zeng G.M., Du C.Y., Tang L., Zhou J.F., Li Z.W. (2008). Spatial analyzing system for urban land-use management based on GIS and multi-criteria assessment modeling. Prog. Nat. Sci. Mater. Int..

[35] Xu Y., Tang Q., Fan J., Bennett S.J., Li Y. (2011). Assessing construction land potential and its spatial pattern in China. Landsc. Urban Plan..

[36] Li S., Zhao X., Pu J., Miao P., Wang Q., Tan K. (2021). Optimize and control territorial spatial functional areas to improve the ecological stability and total environment in karst areas of Southwest China. Land Use Policy.

[37] Yang Y., Chen J., Huang R., Feng Z., Zhou G., You H., Han X. (2022). Construction of Ecological Security Pattern Based on the Importance of Ecological Protection—A Case Study of Guangxi, a Karst Region in China. Int. J. Environ. Res. Public Health.

[38] Wei Y., Wang R., Zhuo X., Feng H. (2021). Research on Comprehensive Evaluation and Coordinated Development of Water Resources Carrying Capacity in Qingjiang River Basin, China. Sustainability.

[39] Kreuter U.P., Harris H.G., Matlock M.D., Lacey R.E. (2001). Change in ecosystem service values in the San Antonio area, Texas. Ecol. Econ..

[40] Ng C.N., Xie Y.J., Yu X.J. (2013). Integrating landscape connectivity into the evaluation of ecosystem services for biodiversity conservation and its implications for landscape planning. Appl. Geogr..

